# Postsynthetic Photocontrol of Giant Liposomes via Fusion-Based Photolipid Doping

**DOI:** 10.1021/acs.langmuir.2c01685

**Published:** 2022-09-21

**Authors:** Stefanie D. Pritzl, Johannes Morstein, Sophia Kahler, David B. Konrad, Dirk Trauner, Theobald Lohmüller

**Affiliations:** † Chair for Photonics and Optoelectronics, Nano-Institute Munich, Department of Physics, Ludwig-Maximilians-Universität (LMU), 80539 Munich, Germany; ‡ Department of Chemistry, New York University, Silver Center, New York 10003, United States; § Department of Cellular and Molecular Pharmacology, UCSF, San Francisco, California 94143, United States; ∥ Department of Pharmacy, Ludwig-Maximilians-Universität (LMU), 81377 Munich, Germany

## Abstract

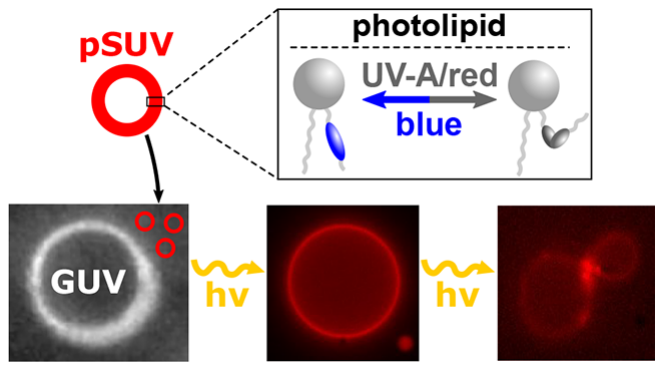

We report on photolipid doping of giant unilamellar vesicles (GUVs) *via* vesicle fusion with small unilamellar photolipid vesicles (pSUVs), which enables retroactive optical control of the membrane properties. We observe that vesicle fusion is light-dependent, if the phospholipids are neutral. Charge-mediated fusion involving anionic and cationic lipid molecules augments the overall fusion performance and doping efficiency, even in the absence of light exposure. Using phosphatidylcholine analogs with one or two azobenzene photoswitches (*azo*-**PC** and **d***azo-***PC**) affects domain formation, bending stiffness, and shape of the resulting vesicles in response to irradiation. Moreover, we show that optical membrane control can be extended to long wavelengths using red-absorbing photolipids (***red***-*azo*-**PC**). Combined, our findings present an attractive and practical method for the precise delivery of photolipids, which offers new prospects for the optical control of membrane function.

## Introduction

Photoswitchable lipids (“photolipids”) are versatile molecular nanoagents for controlling lipid-mediated processes and membrane properties with light.^1^ Phosphatidylcholine derivatives bearing a photoswitchable azobenzene group in one of their hydrocarbon tails^2−5^ have been used to control lipid diffusion,^6^ domain formation,^3^ bilayer rigidity,^2^ permeability,^7^ and protein molecular diffusion^8^ in synthetic bilayer membranes. However, in all of these examples, the photolipids were already added during the preparation of the vesicles and lipid bilayer assemblies. For many applications in synthetic biology or pharmacology, the postdoping of photolipids into an already formed bilayer membrane would be highly desirable to take advantage of the full potential offered by photolipids to control membrane properties. So far, amphiphilic or membrane-targeted photoswitchable molecules and fatty acids^9^ have been used in the context of the cell membrane and protein modification, allowing one to modulate neuronal firing,^10^ ion channel excitability,^11^ and cell signaling.^12−14^

Phospholipid doping and vesicle fusion can in principle be achieved by modulating the lipid synthesis pathway or by introducing specific phospholipid molecules *via* vesicle fusion or lipid uptake from solution. While the incorporation of photolipids *via* synthesis was recently achieved,^15^ this is not applicable to artificial systems lacking the required enzymatic pathways. Light-dependent and retroactive doping could present a highly attractive alternative and modular strategy to render membranes photoswitchable.

A number of chemical and physical strategies for introducing synthetic phospholipids into lipid membranes have been demonstrated in recent years, including the use of fusogenic proteins^16^ and ligands,^17^ osmotic gradients,^18^ electrofusion,^19^ or charge-mediated fusion.^20,21^ In the case of photolipids, another possibility is added since the photoswitching process itself can aid the fusion process. Suzuki et al.^22^ showed that azobenzene surfactants could increase the tension of bilayer membranes, which facilitates photocontrolled fusion of cell-sized vesicles. For photolipid membranes, Scheidt et al.^23^ reported that light-triggered lipid splay, attributed to the conformational change between the *trans* and *cis* forms, plays a causal role in vesicle coalescence. Successful membrane mixing was observed during UV exposure and *trans*-to-*cis* isomerization using vesicles that contained up to 20 mol % of photolipids. Similar observations were reported by Morgan et al.^20^ In this case, the authors showed that vesicles of binary dipalmitoylphosphatidylcholine (DPPC)/photolipid mixtures can interact with dye-loaded gel-phase DPPC vesicles leading to enhanced permeability and dye release only after the photolipids were subject to UV photolysis. However, postsynthetic photolipid doping into fluid cell-sized vesicle membranes and subsequent reversible photocontrol of the membrane mechanics and bilayer order has not been reported to date. To achieve this aim, two factors have to be considered. On the one hand, a suitable strategy to introduce enough photolipids into bilayer membranes is required to obtain an appreciable photoswitching effect on membrane properties. On the other hand, the photolipids have to display a significant conformation change to achieve a strong impact on the bilayer properties.

In this work, we investigate the possibility for retroactive photolipid doping in synthetic bilayer membranes by comparing two fusion strategies with three different types of photolipids. For this, we compare photoinduced and charge-mediated fusion with photolipids that have an azobenzene group in either one (*azo*-**PC**) or both (**d***azo*-**PC**) hydrocarbon tails. We observe that photolipid uptake in 1,2-dioleoyl-*sn*-glycero-3-phosphocholine (DOPC) vesicles by photoisomerization is possible in both cases, although charge-mediated fusion *via* cationic and anionic lipids further enhances the overall fusion process to obtain photocontrol of membrane properties. Importantly, we demonstrate that the properties of photolipid-doped giant unilamellar vesicles (GUVs) can be controlled with light after the fusion. Photoisomerization of *azo*-**PC** allows one to reversibly affect the membrane order and phase separation, while **d***azo*-**PC** doping enables optical control of membrane fluctuations and vesicle shape. In addition, we show that charge-mediated fusion is also adequate to postsynthetically dope the red-shifted photolipid ***red***-*azo*-**PC** into regular GUVs and obtain subsequent photocontrol of the vesicle stiffness. These ***red***-*azo*-**PC** lipids can be switched with tissue-penetrating red light, which increases the biocompatibility for *in vivo* studies. Overall, our findings pave the way for applications in cell membrane systems as the doping of lipid molecules *via* vesicle fusion is a viable and compatible strategy in a cellular context.^24^

## Experimental Section

### Preparation of Small Unilamellar Photolipid Vesicles (pSUVs)

We prepared pSUVs by tip sonication as previously reported.^6^ Briefly, 100 μL of photolipids (*azo*-**PC** or **d***azo*-**PC)** dissolved in chloroform (amylene stabilized, Merck) at 6.36 mM were mixed with 1 mol % TexasRed-DHPE (TexasRed 1,2-dihexadecanoyl-*sn*-glycero-3-phosphoethanolamine, Thermo Fisher) and dried by air to deposit a lipid film on the bottom of a glass flask. The lipid film was then rehydrated with 1.5 mL of deionized water. Next, the solution was tip sonicated on ice twice for 30 s (Bandelin, Sonopuls) and centrifuged for 10 min with a relative centrifugal force (RCF) of 35.8 rpm^2^m before being stored at 4 °C until further use. Fluorescent ***red***-*azo*-**PC** pSUVs were prepared from 99 mol % ***red***-*azo*-**PC** and 1 mol % Atto633-DPPE (Atto633 1,2-dipalmitoyl-*sn*-glycero-3-phosphoethanolamine, Atto-Tec). Cationic pSUVs contained 3 mol % of the positively charged lipid DOTAP (1,2-dioleoyl-3-trimethylammonium-propane, Avanti Polar Lipids). The overall molar contents of these pSUVs were 94 mol % photolipids, 3 mol % DOTAP, and 1 mol % dye-labeled lipids. We again used TR-DHPE or Atto633-DPPE to label cationic *azo*-**PC** and **d***azo*-**PC** pSUVs or ***red***-*azo*-**PC** pSUVs, respectively.

### Preparation of Giant Unilamellar Vesicles

We prepared GUVs as previously reported in a home-built device based on the electroformation method.^3^ To synthesize label-free GUVs, DOPC (1,2-dioleoyl-*sn*-glycero-3-phosphocholine, Avanti Polar Lipids) lipids were first dissolved in chloroform at a concentration of 6.36 mM. For experiments on charge-mediated fusion, 5 mol % of PA (1,2-distearoyl-*sn*-glycero-3-phosphate, Avanti Polar Lipids) were added. An amount of 5 μL of the solution was spread with a syringe on two platinum wires which are 3 mm apart and span a Teflon chamber. Then, 1.5 mL of a sucrose solution (0.3 M), enough to cover the wires, was added. The chamber was heated to 70 °C, and an actuated electric field (10 Hz, 3 V) was applied for 120 min to the wires. The 0.3 M solution containing the GUVs was stored at room temperature for further use. No further buffering components or salts were added. To facilitate microscopy measurements, the GUV solution was diluted with a 0.3 M glucose solution (1:1 ratio GUV solution/glucose solution) to induce sedimentation of the GUVs at the sample bottom.

### Microscopy

Sample imaging was done on an inverted microscope (IX81, Olympus) with a 100× objective (NA = 1.35, UPlanSApo, Olympus) in epifluorescence configuration. For epifluorescence microscopy, an HBO lamp was used, and imaging as well as excitation of the respective photolipid isomers were realized with suitable filter cubes that are a blue filter set (λ_exc_: 470–490 nm, λ_em_ > 520 nm), a green filter set (λ_exc_: 510–550 nm, λ_em_ > 590 nm), a UV filter set (λ_exc_: 330–385 nm, λ_em_ > 420 nm), and a red filter set (λ_exc_: 623/32 nm, λ_em_ > 680/42 nm). To prevent unwanted switching and photobleaching, suitable optical filters were utilized. In addition, reflectance interference contrast microscopy (RICM) was used to visualize label-free GUVs. RICM was achieved using a filter cube containing a 50:50 beamsplitter (PBSW-532R, Thorlabds) in the dichroic position and a narrow bandpass filter (FL532-10, Thorlabs) to select the excitation wavelength, λ_exc_ = 532 nm. The vesicle samples were imaged with a Canon EOS 6D or a CCD camera (iXon Ultra, Andor). The photoluminescence (PL) intensities of dye-containing vesicle contours were determined by summing the intensities *I* of all pixels *p*_*i*,*j*_ within a circle of radius *R*_circle_ = 4*R*_vesicle_/3 around the center of the vesicle of each image frame according to
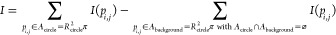
The subtrahend serves for background correction.

## Results and Discussion

### Photolipid Molecules and Vesicles

**Figure 1:**
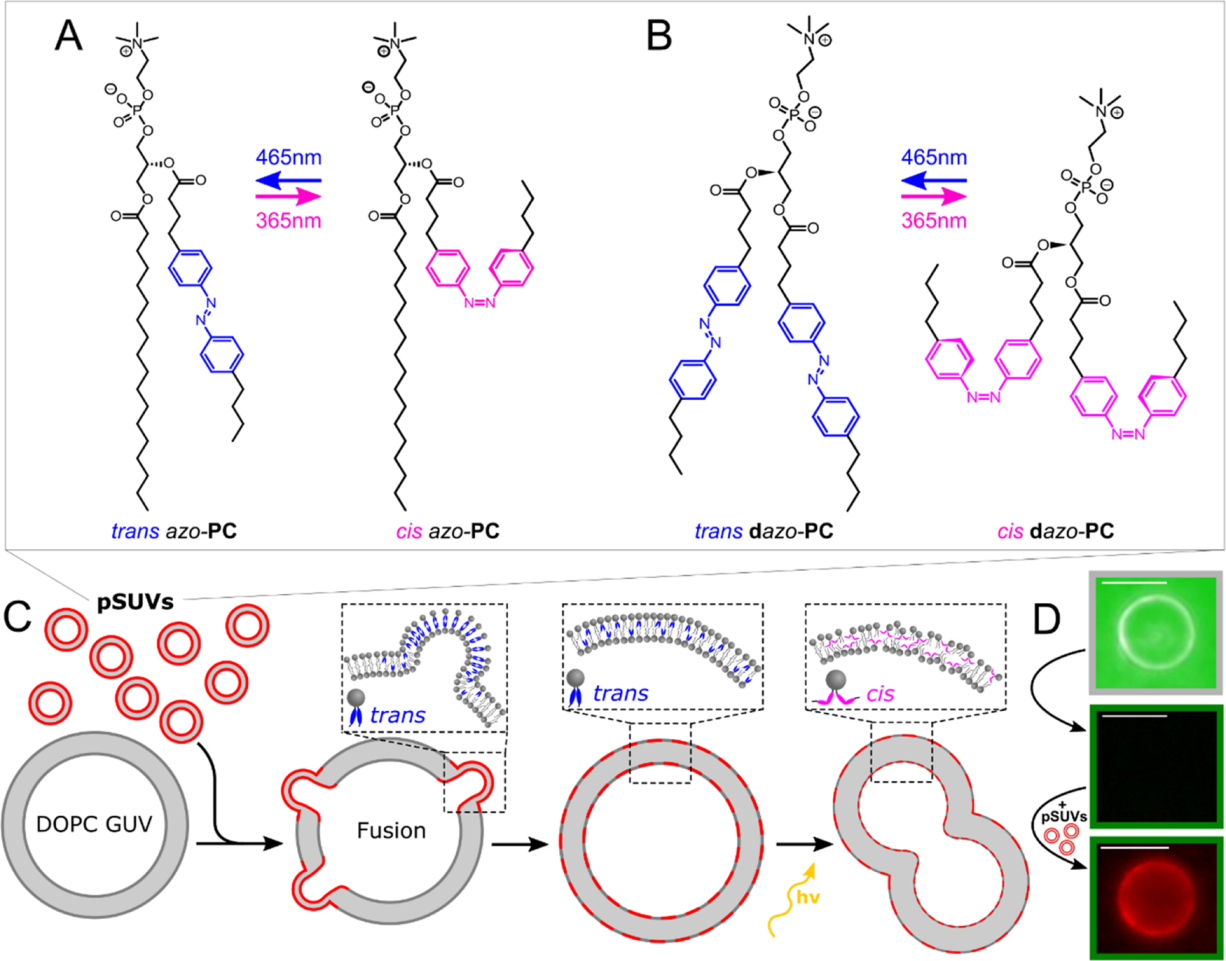
Schematic of the vesicle fusion assay. Red-fluorescent pSUVs with either *azo*-**PC** (A) or **d***azo*-**PC** (B) are mixed with label-free DOPC GUVs. Photoswitching alters the membrane properties leading to e.g., vesicle budding in the presence of *cis*-**d***azo*-**PC** photolipids (C). Upon pSUV uptake, the GUV becomes fluorescent (D). Top: RICM image of an unlabeled DOPC GUV. Middle: No fluorescence signal is obtained from the pure DOPC GUV. Bottom: Incubation with TR-labeled pSUVs for ∼5 min renders the GUV fluorescent due to vesicle fusion. Fluorescence images were taken under equal acquisition conditions (λ_exc_: 510–550 nm, λ_em_ > 590 nm). Scale bars: 10 μm.

The photolipid *azo*-**PC**, which contains an azobenzene unit in the *sn*2 tail, was synthesized according to a previous protocol.^3^ Photoswitching between the isomeric forms, *trans*- and *cis-azo*-**PC**, is achieved with blue and UV-A light, respectively (Figure 1A). The **d***azo*-**PC** lipids were synthesized over two steps following a new synthetic method for the preparation of sufficiently large amounts of photolipids required for elaborate biophysical studies (Figure 1B, Supporting Information, S1). In the first step, **AzoLPC** bearing a single azobenzene group was prepared by selective monoacylation of l-α-glycerylphosphorylcholine (α-GPC) in 30% yield. In the second step, the photoswitchable fatty acid **FAAzo-4** was installed at the *sn*2 position *via* a Yamaguchi esterification, and **d***azo*-**PC** was obtained with an overall yield of 27%. In the dark-adapted state (prior to any photoswitching), **d***azo*-**PC** assumes the thermally favored *trans* conformation. NMR measurements revealed a *trans*/*cis* ratio of 99:1 (Supporting Information, S2). For *azo*-**PC** photolipids, a *trans*/*cis* ratio of 100:0 was reported.^25^ As shown for regular *azo*-**PC**, the newly synthesized **d***azo*-**PC** lipids can also be switched between the *trans* and *cis* form using UV-A (*trans*/*cis* = 2:98) and blue light (*trans*/*cis* = 79:21) over many switching cycles without a sign of sample decomposition (NMR data and absorbance spectra are included in the Supporting Information, Figure S2A,B). Moreover, the switching kinetics of monomeric *azo*-**PC** and **d***azo*-**PC** (free lipids dissolved in CHCl_3_) are similar, while those of *azo*-**PC** and **d***azo*-**PC** pSUVs differ by a factor of ∼2 (Supporting Information, Figure S2C), which is similar to previous studies of azobenzene-containing surfactants and micelles.^26^

We performed DLS measurements of **d***azo*-**PC** pSUVs to study liposome stability (Supporting Information, Figure S3). The average diameters of **d***azo*-**PC** pSUVs increase from (264 ± 6) to (313 ± 11) nm upon photoswitching from *trans* to *cis*, which corresponds to an average size change of (15.6 ± 1.1)%. In comparison, a reversible size change of only ∼3% was reported for *azo*-**PC** pSUVs.^2^ The vesicles could be switched over several cycles, indicating that both *trans*- and *cis*-**d***azo*-**PC** retain stable vesicles.

### Photo-Triggered Vesicle Fusion

We studied the possibility to dope synthetic lipid membranes with photolipid molecules by fusing pSUVs labeled with 1 mol % of TexasRed-DHPE into nonfluorescent DOPC GUVs during sequential photoswitching (Figure 1C). The uptake of photolipids in the GUV membrane is observed indirectly by fluorescence microscopy since TR-lipids along with the photolipids are accumulated in the GUV membrane due to the fusion process (Figure 1D). Prior to the addition of the pSUVs, we mixed the GUV sample with glucose solution (300 mM) to enable imaging of sedimented vesicles. Unlabeled GUVs were identified by RICM, as exemplarily shown in Figure 1D (top image). Prior to adding the pSUVs, the GUVs did not exhibit any fluorescence upon excitation with 510–550 nm light (Figure 1D, middle image). After incubating the sample with TR-labeled pSUVs for ∼5 min, the GUV membrane displayed homogeneous fluorescence, indicative of the uptake of the TR-dye *via* vesicle fusion (Figure 1D, bottom image).

Fluorescence video microscopy measurements were performed using a green (510–550 nm) and a UV-A (330–385 nm) filter cube. These illumination conditions were chosen to allow for imaging and photolipid isomerization at the same time. Not only is TR-DHPE excitable with UV-A and green light (Supporting Information, Figure S4), but also the photolipids are efficiently switched between *trans* and *cis* using 365 nm and 550 nm light (Supporting Information, S5).

**Figure 2:**
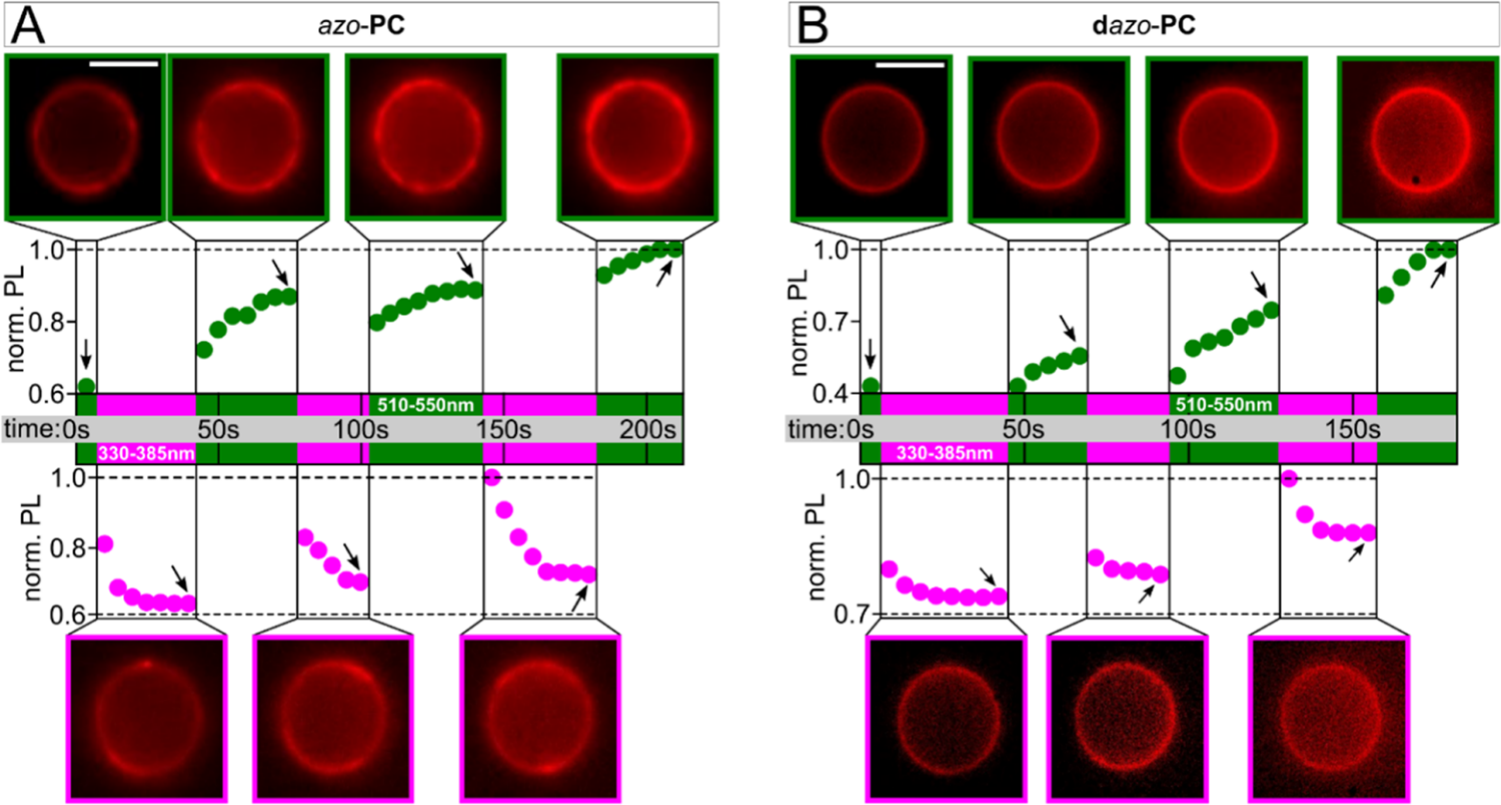
Photo-triggered vesicle fusion over time. For *azo*-**PC** (A) and **d***azo*-**PC** (B), the PL intensities of the vesicle contours increase during green-light exposure (*cis*-to-trans isomerization; green boxes/dots), which is the result of pSUVs fusing with the GUV membrane. For UV illumination (pink boxes/dots), the fluorescence intensity drops at first due to TR fluorescence quenching by the *cis* photolipids, until a steady state is reached, indicating that the fusion process with *cis* pSUVs is not efficient. The vesicle images represent the data points that are marked by the black arrows. The time “0 s” represents the starting point of the fusion assay with consecutive *cis*/*trans* photoisomerization. Scale bars: 10 μm.

The samples were sequentially illuminated with green and UV-A light, and we analyzed the fluorescence intensities over several switching cycles. Prior to the experiment, the TR-labeled pSUVs were stored in the dark to convert them to the thermodynamically stable *trans* form. After mixing the pSUVs with DOPC vesicles, the sample was first illuminated with green light under the microscope to adjust the focus and acquisition settings. This results in prefusion of a tiny fraction of pSUVs, which is sufficient to locate individual GUVs. We then defined *t* = 0 as the starting point for the switching cycles (Figure 2).

The sequential pSUV uptake was quantified by determining the fluorescence intensity increase of the vesicle contours. During green-light exposure, the photoluminescence (PL) intensities increase exponentially. The average PL rise times are (18.0 ± 3.7) s and (14.7 ± 4.8) s for *azo*-**PC** and **d***azo*-**PC**, respectively. For UV-A illumination, an immediate exponential decrease of the fluorescence intensities by 5–30 % is observed that saturates after ∼20 s. The mean PL decay times are (14.4 ± 7.3) s and (4.2 ± 0.6) s for *azo*-**PC** and **d***azo*-**PC**, respectively.

This PL decay can be attributed to the stronger TR fluorescence quenching by the *cis* compared to the *trans* isomer. In general, azobenzenes are well-known dark quenchers for certain fluorophores, and such effects must be considered for the analysis of the fluorescence microscope images.^27^ In azobenzene-containing aggregates, isomer-specific photomodulation is observed, where *cis* azobenzenes generally induce a larger decrease in the fluorophore emission.^28,29^ To account for photomodulation by the two isomers, we quantified the PL intensities of *azo*-**PC** and **d***azo*-**PC** pSUVs, which contained 1 mol % of TR-DHPE (Supporting Information, S6). In both samples, the TR emission intensities are lower in the presence of *cis* isomers compared to *trans* photolipids (Supporting Information, Figure S6). The photomodulation efficiencies are 79% and 72% for *azo*-**PC** and **d***azo*-**PC**, respectively.

Taking a closer look at the PL curves of the fusion experiment shown in Figure 2, one finds that the intensity increases measured for green-light exposure are almost continuous, only interrupted by the UV illumination steps. The average TR emission intensity differences prior (PL^prior^) and after (PL^after^) UV-A light exposure, i.e., ⟨Δ*I*_*i*_⟩ = ⟨PL_*i*_^prior^ – PL_*i*_^after^⟩ with *I* = 3 representing the number of switching cycles, are only (7 ± 3)% and (2 ± 3)% for *azo*-**PC** and **d***azo*-**PC**, respectively. This suggests that fusion with pSUVs is enhanced during *cis*-to-*trans* isomerization, while the reverse direction plays a minor role. Furthermore, vesicle fusion occurs only during lipid isomerization and slows down or stops when a *cis*/*trans* equilibrium is reached. Notably, the positive average difference (⟨Δ*I*_*i*_⟩ > 0) further indicates that photobleaching is negligible. This is also supported by the saturation of the PL decrease during UV-A exposure at a constant intensity level. This observation was further confirmed by control experiments, where green-light illumination was extended over a longer time period (Supporting Information, Figure S7). No further increase in the fluorescence intensity was observed in this case, indicating that membrane reorganization due to changes in lipid conformation is important for the fusion process. For *cis*-to-*trans* isomerization, bilayer defects are introduced due to the rapid reduction of the cross-sectional area of *azo*-**PC**.^7^ Such defects or membrane voids have been reported as a driving force for membrane fusion.^30^ Transient pore formation during *cis*-to-*trans* switching was observed in pure photolipid membranes, while permeability after *trans*-to-*cis* isomerization was found for lipid mixtures and binary membrane compositions with only a 2–12 mol % amount of photolipids.^31−34^ This indicates that the formation of membrane voids is strongly dependent on the amount of photolipids, which is also in agreement with the recent work by Scheidt et al.^23^ demonstrating UV-A-triggered fusion between liposomes made of POPC or DOPC and up to 20 mol % *azo*-**PC**.

However, the efficiency of light-triggered vesicle fusion was not sufficient to obtain visible photocontrol over membrane properties of photolipid-doped GUVs, even after extending the fusion time to 30 min. The vesicles showed no sign of membrane fluctuations, domain formation, shape transformations, or any other form of photoinduced effects that were observed for pure photolipid vesicles, suggesting that not enough photolipids were fused into the GUV bilayer by light-mediated fusion alone.

### Charge-Mediated Vesicle Fusion

To enhance the photolipid uptake, we followed a second strategy, where we added anionic and cationic lipids to enable charge-mediated fusion *via* attractive electrostatic interactions and subsequent membrane adhesion and coalescence.^21^ First, the viability of this approach was tested with regular nonswitchable vesicles. We prepared anionic DOPC GUVs with 5 mol % of the negatively charged phospholipid PA (1,2-distearoyl-*sn*-glycero-3-phosphate) and cationic, fluorescent DOPC SUVs with 3 mol % of the positively charged lipid DOTAP. While PA itself favors fusion due to its cone shape and subsequent negative curvature,^35^ DOTAP, on the other hand, becomes fusogenic in the presence of neutral colipids like e.g., DOPE and DOPC.^36,37^ Mixing the oppositely charged SUVs and GUVs resulted in vesicle fusion (Supporting Information, Figure S8).

**Figure 3:**
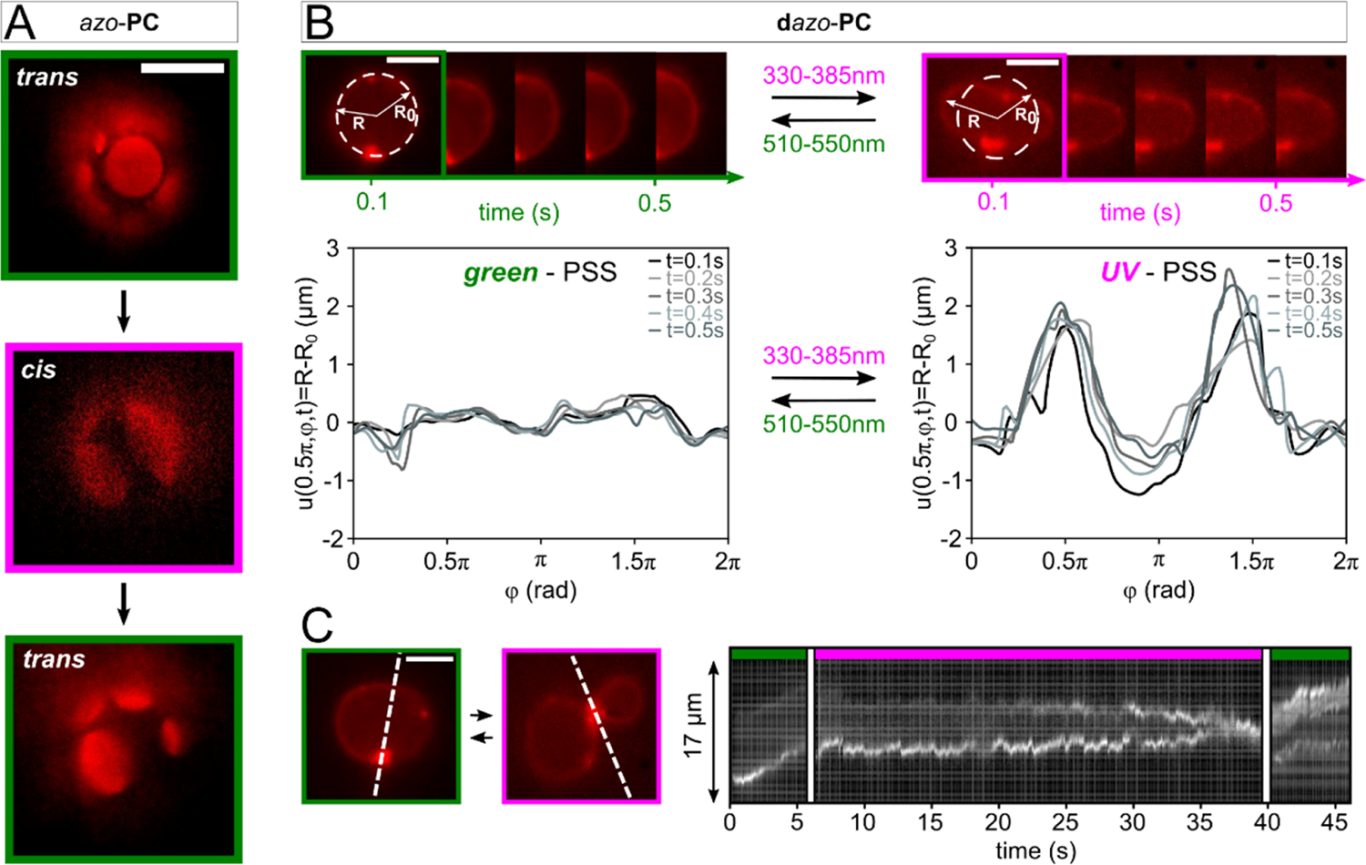
Optical control of photolipid-doped GUVs. (A) Fluorescence images of *azo*-**PC**-doped GUV. The box colors represent the illumination wavelengths, i.e., green light of 510–550 nm (green boxes) and UV-A light of 330–385 nm (pink boxes). For both illumination conditions, dark and bright areas are visible on the vesicle contour, indicating phase separation in the GUV bilayer. (B) Fluorescence images of **d***azo*-**PC**-doped GUV at different time steps. The vesicle shape changes from sphere-like to elliptical upon isomerization with UV-A (pink) and green (green) light. The vesicle contour plots illustrate the change of the membrane fluctuations and vesicle shape. (C) UV-A exposure for 32 s results in a budding transition. Green-light illumination restores the initial spherical shape, which is highlighted in the kymograph. Scale bars: 10 μm. The GUVs were composed of 95 mol % DOPC and 5 mol % PA. The SUVs contained 96 mol % photolipids (*azo*-**PC** or **d***azo*-**PC**), 1 mol % TR-DHPE, and 3 mol % DOTAP.

We then prepared cationic fluorescent pSUVs with 3 mol % DOTAP and added them to a solution of anionic DOPC GUVs (5 mol % PA). After mixing, the GUVs were imaged under the microscope using green light (Figure 3). The uptake of *azo*-**PC** vesicles immediately led to dark and bright areas of the GUV membrane, suggesting phase separation of the DOPC and *azo*-**PC** lipids (Figure 3A). Vesicles of binary compositions containing *azo*-**PC**/DPhPC or *azo*-**PC**/DOPC have been suggested to feature domains in the presence of *trans* photolipids due to dipolar interactions and H-aggregate formation, while no phase separation was observed for *cis* photolipids.^3^ Domain formation can be further enhanced by the presence of the two oppositely charged lipids DOTAP and PA. Vequi-Suplicy et al.,^38^ for example, have reported that the fusion of DOTAP-labeled liposomes with anionic ones results in domain formation of the oppositely charged lipid species.

We changed the illumination conditions to UV-A exposure to convert the photolipids to the *cis* form and found that the domains started to merge, while upon back switching to *trans*, they disassembled again into smaller domains. The average number (**#**) and area (*DA*) of the GUV domains (Figure 3A) changed between #_*trans*_ = (8 ± 2) and **#**_*cis*_ = (3 ± 1), and *DA*_*trans*_ = (55.6 ± 13.3)% and *DA*_*cis*_ = (72.6 ± 16.9)%. The values represent the mean and the standard deviation of two switching cycles, respectively. The number of domains and the domain sizes, i.e., their surface areas, were determined from a three-dimensional (3D) image that was reconstructed from multiple two-dimensional (2D) epifluorescence images (Supporting Information, Movie S1). All six vesicles studied displayed phase separation and domain formation upon *azo*-**PC** pSUV uptake. These findings hint at a fractional lipid reorganization during photoswitching, which could be a consequence of charged lipid domains preventing complete mixing of the *cis* photolipids and DOPC. Binary *trans*-*azo*-**PC**/DOPC vesicles have been suggested to display nanodomains, while lipid mixing occurs in *cis-azo*-**PC**-containing vesicles.^39^ The charged lipid species can aid domain formation in the presence of *trans-azo*-**PC** leading to the observed micrometer-sized domains.

For charge-mediated **d***azo*-**PC** doping, we found that vesicle fusion even allows to gain control over membrane fluctuations and vesicle shape transformations, owing to the presence of photoswitches in both lipid tails (Figure 3B,C). The **d***azo*-**PC**-doped GUV shown in Figure 3B already exhibits a slightly elliptical shape during green-light exposure. Switching to UV-A light results in the appearance of membrane fluctuations within milliseconds and further elongation (Supporting Information, Movie S2). Furthermore, budding events were observed for 48% of the GUVs after prolonged UV-A exposure (Figure 3C; Supporting Information, Movie S3). Back-switching to *trans* with green light allowed for reversing the shape transition and recouping a spherical contour (Supporting Information, Movie S4). Although the process was repeatable, a clear correlation between UV-A exposure time and budding events was not observed. We analyzed the membrane undulations of this GUV in more detail by determining the vesicle contours as a function of the polar angle. An increase of the mean (= averaged mean of single spectra in Figure 3C) fluctuation intensities from (0.01 ± 0.27) to (0.07 ± 0.88) μm is observed when switching from *trans* to *cis*. Stronger membrane fluctuations in the *cis* state are thereby indicative of a lower vesicle bending stiffness.

We confirmed this by control measurements where we analyzed the membrane fluctuations of pure **d***azo*-**PC** GUVs using a previously reported protocol^40^ (Supporting Information, S9). We found an average bending stiffness increase from κ_*trans*_ ∼ 10^–20^ J to κ_*cis*_ ∼ 10^–17^ J, which was previously reported to be in the range of gel-to-fluid-like membrane phase transitions.^41^ Notably, long integration times (200 ms) are required for vesicle contour analysis in our experiment. The membrane bending rigidity could therefore not be analyzed with the same level of accuracy typically obtained in membrane fluctuation spectroscopy.^42^ The photosensitive nature of the photolipids demands careful control of the illumination conditions to avoid any unintentional switching. Label-free techniques such as e.g., phase-contrast or darkfield microscopy are therefore not suitable due to their extended UV/vis imaging range. Instead, we relied on fluorescence microscopy with dyes that are excitable at the same wavelengths as the photoswitch itself. While TR-DHPE is excitable with UV-A light, it only shows a low absorbance and therefore low emission, which requires rather long integration times for fluorescence imaging (Supporting Information, Figure S4). For comparison, short integration times of <10 ms are typically applied in fluctuation spectroscopy to account for thermal undulations and to assess membrane bending rigidity with high accuracy.^42^ However, even with these experimental constraints, our measurements clearly show that the bending rigidity of pure **d***azo*-**PC** GUVs and subsequently also of photolipid-doped GUVs can change significantly. To further support this conclusion, we performed fluorescence recovery after photobleaching (FRAP) experiments and determined the diffusion coefficients of supported **d***azo*-**PC** bilayers in the *trans* and *cis* states that were labeled with 1 mol % TR-DHPE (Supporting Information, S10). The bilayers displayed average diffusion coefficients of *D*_*trans*_ = (0.11 ± 0.01) μm^2^ s^–1^ and *D*_*cis*_ = (1.3 ± 0.1 μm^2^ s^–1^), which, again, agrees well with a typical increase of membrane fluidity from gel-like to fluid.^43,44^

In total, 48% of 25 studied GUVs exhibited budding events, while 16% showed at least enhanced membrane fluctuations and 8% displayed further vesicle shape deformations such as vesicle splitting and pearling transitions upon *trans*-to-*cis* isomerization (Supporting Information, S11). The bright spot at the neck region of the vesicle budding event shown in Figure 3C indicates a local accumulation of TR at the GUV surface. Since the GUVs themselves were not labeled, these dyes stem from the pSUVs that were added to instigate vesicle fusion. Steinkühler et al.^45^ have reported that protein binding to GUVs can control the spontaneous membrane curvature, leading to budding and eventually even splitting of single vesicles into daughter cells. Potentially, the observed accumulation of lipid dyes or adsorbed pSUVs on the outer GUV surface could therefore facilitate vesicle budding as well, in particular in combination with the membrane stiffness modulation obtained by photoswitching (Supporting Information, S9).

### Photolipid-Dependent Fusion Efficiency

For *azo*-**PC**-containing GUVs, we previously suggested that the addition of DOPC can lead to phase separation and domain formation in the binary *trans-azo*-**PC**/DOPC vesicles.^3^**D***azo*-**PC**, however, can exhibit phase separation also in the absence of a second lipid species, which we observed for GUVs only containing **d***azo*-**PC** and 1 mol % TR-DHPE (Supporting Information, Figure S12). This raises the question of why the GUVs do not display domains after vesicle fusion and **d***azo*-**PC** accumulation, as is the case for *azo*-**PC** (Figure 2). A possible explanation could be a lower fusion efficiency of **d***azo*-**PC** than of *azo*-**PC**. To support this hypothesis, we applied a fluorescence-based fusion assay and mixed the cationic red-fluorescent pSUVs with DOPC GUVs that not only contained PA but also green-fluorescent Atto465-DOPE (Supporting Information, S13). After fusion, the doped GUVs displayed both colors, red and green (Supporting Information, Figure S13). The fluorescence intensities of the two dyes changed in response to the lipid uptake. We compared the ratios of these emission intensities with calibrated PL ratios, which we derived from fluorescence measurements of vesicle samples where the lipids were already added during the liposome preparation. These samples contained defined amounts of TR-DHPE, Atto465-DOPE, PA, DOTAP, DOPC, and *azo*-**PC** or **d***azo*-**PC** to resemble the fusion process and reflect various fusion efficiencies and photolipid doping levels. The measurements indicate that the average photolipid uptakes are (40 ± 30)% for *azo*-**PC** and (20 ± 15)% for **d***azo*-**PC**, which shows that *azo*-**PC** uptake is more effective.

### *Red*-*azo*-PC Doping

**Figure 4:**
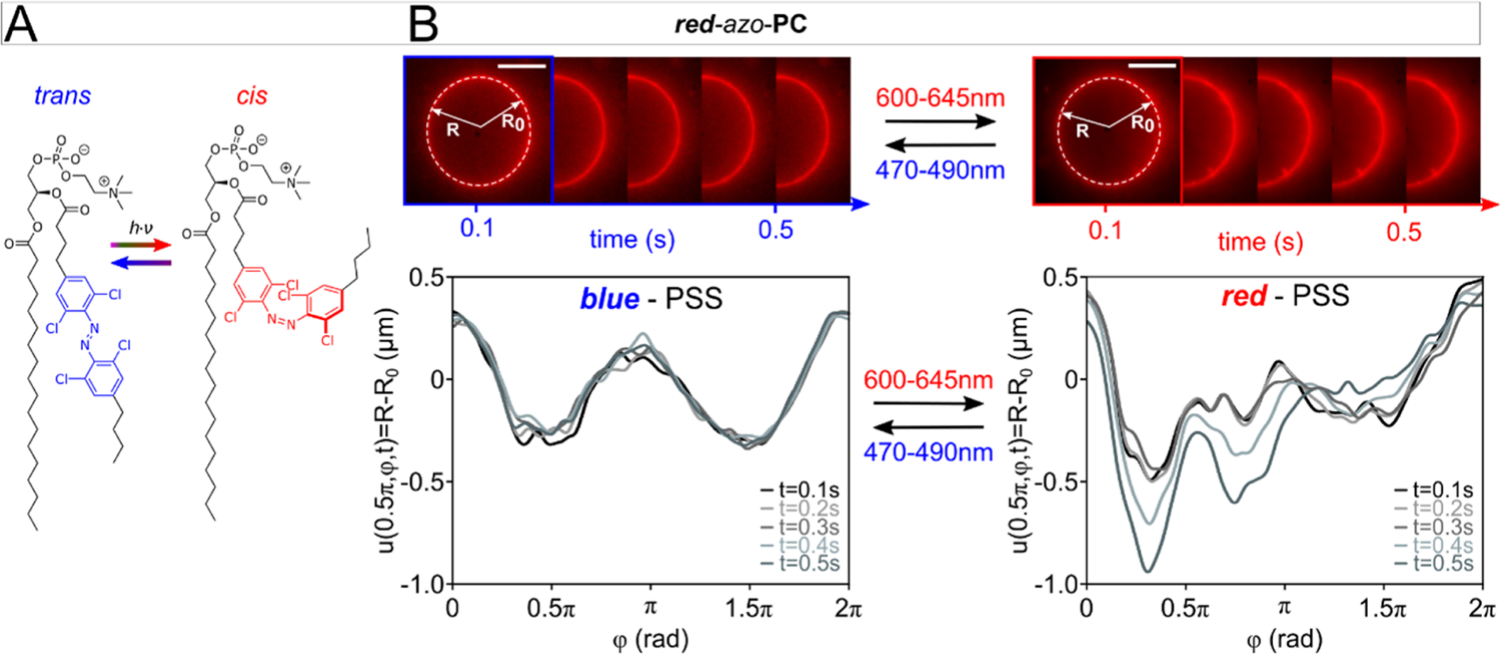
Doping with ***red***-*azo*-**PC**. (A) ***Red***-*azo*-**PC** can be switched between the *trans* and *cis* isomer using green/red and purple/blue light, respectively. (B) Vesicle images after the uptake of ***red***-azo-**PC** upon blue- and red-light exposure. The contour plots represent the membrane fluctuations in the photostationary state (PSS) reached with blue (left) and red (right) light, respectively. Scale bars: 10 μm.

As a perspective for future applications in biological systems, we also tested the fusion assay with the red-shifted photolipid ***red***-*azo*-**PC** that contains a tetra-*ortho*-chlorinated azobenzene unit in the *sn*2 tail (Figure 4A).

The *ortho*-substitution induces a spectral shift of the isomerization wavelengths rendering photoswitching with blue and red light possible.^40^ This is of particular advantage for biological applications since red light is less absorbed by tissue than UV-A light allowing for deeper penetration depths.^46^ The pSUVs were prepared from 96 mol % ***red***-*azo*-**PC**, 1 mol % Atto633-DPPE, and 3 mol % DOTAP. Prior to use, they were again stored in the dark to convert them to a photostationary state (PSS) with mostly *trans* lipids. After mixing the pSUVs with the nonfluorescent anionic DOPC GUVs, we initially imaged the doped vesicles with blue light using a blue filter cube (470–490 nm) to maintain the *trans*-rich state (Figure 4B). We then changed to red-light (600–645 nm) illumination to induce the *trans*-to-*cis* isomerization. The vesicle contour started to immediately fluctuate (Supporting information, Movie S5). These fluctuations were again studied in more detail by determining the vesicle contour profiles as a function of the polar angle. The membrane fluctuations displayed in Figure 4B increase by a mean factor of ∼2.7 from (0.7 ± 2.1) to (1.7 ± 3.5) μm, which corresponds to the mean value and standard deviation of an averaged spectrum derived from the five single spectra, when switching the red-shifted lipids from *trans* to *cis*. This result agrees well with photo-triggered membrane undulations in pure ***red***-*azo*-**PC** GUVs^40^ and shows that the reached ***red***-*azo*-**PC** doping level is sufficient to gain optical control of the membrane mechanics retroactively *via* fusion of charged vesicles. Overall, 83% of the GUVs showed enhanced membrane fluctuations upon ***red***-*azo*-**PC** uptake and *trans*-to-*cis* isomerization. This might seem surprising, considering that the doping of *azo*-**PC**, which also contain a single azobenzene unit in the *sn*2 lipid tail, results in the formation of μm-sized domains. However, *trans*-*azo*-**PC** and **-d***azo***-PC** photolipids display H-aggregate formation when assembled to bilayer membranes (Supporting Information, S2). This is not the case for ***red***-*azo*-**PC**.^40^ Hence, a more homogeneous distribution of the ***red***-*azo*-**PC** photolipids after their uptake can be expected, which is in agreement with the homogeneous coloring of the vesicle membrane contours shown in Figure 4B.

## Conclusions

In conclusion, we have shown that regular GUVs can be efficiently doped with photolipid molecules *via* photocontrolled and charge-mediated vesicle fusion with 3–5 mol % of the charged lipid species DOTAP and PA. A*zo*-**PC** doping leads to a change of membrane organization and domain formation in the GUV bilayer, while **d***azo*-**PC** and ***red***-*azo*-**PC** doping render membrane fluctuations, shape transformation, and vesicle budding events photoswitchable. Altogether, our findings demonstrate that the photocontrol of membrane properties *via* isomerization of azobenzene-based photolipids can be transferred to regular, nonswitchable lipid membranes, which emphasizes the potential of photolipid molecules as optical nanoagents for applications in life science.

## Supporting Information Available

Additional information on the synthetic procedures, materials, methods, and analytical/supporting data (PDF)
Domains in*azo*-**PC**-doped GUV (Movie S1) (AVI)
Photo-triggered membrane fluctuations and elongation of **d***azo*-**PC**-doped GUV (Movie S2) (AVI)
UV-A-triggered budding event of **d***azo*-**PC**-doped GUV (Movie S3) (AVI)
Back-switching (*cis*-to-*trans*) of **d***azo*-**PC**-doped GUV/reverse of the shape transition (budding event) (Movie S4) (AVI)
Photo-triggered membranefluctuations of ***red***-*azo*-**PC**-doped GUV (Movie S5) (AVI)

## Supplementary Material

la2c01685_si_001.pdf

la2c01685_si_002.avi

la2c01685_si_003.avi

la2c01685_si_004.avi

la2c01685_si_005.avi

la2c01685_si_006.avi
