# Supplementary material for: Postsynthetic Photocontrol of Giant Liposomes via Fusion-Based Photolipid Doping
Source: Langmuir. 2022 Sep 21;38(39):11941–9. doi: 10.1021/acs.langmuir.2c01685 (PMC9536078; doi:10.1021/acs.langmuir.2c01685)
Supplement: Supplementary file 1 — la2c01685_si_001.pdf [file la2c01685_si_001.pdf]

Supporting Information

Postsynthetic Photocontrol of Giant Liposomes via Fusion-Based Photolipid Doping

Stefanie D. Pritzl [1,†,], Johannes Morstein [2,3], Sophia Kahler [2], David B. Konrad [4], Dirk Trauner [2,†], and Theobald Lohmüller [1,*]*

[1] Chair for Photonics and Optoelectronics, Nano-Institute Munich, Department of Physics, Ludwig-Maximilians-Universität (LMU), 80539 Munich, Germany
[2] Department of Chemistry, New York University, Silver Center, New York 10003, United States
[3] Department of Cellular and Molecular Pharmacology, UCSF, San Francisco, California, United States
[4] Department of Pharmacy, Ludwig-Maximilians-Universität (LMU), 81377 Munich, Germany

Present Addresses:

† (S.D.P.) Department of Physics and Debye Institute for Nanomaterials Science, Utrecht University, Princetonplein 1, 3584 CC Utrecht, The Netherlands

† (D.T.) Department of Chemistry, University of Pennsylvania, Philadelphia, PA 19104-6323, United States

Email: s.d.pritzl@uu.nl, t.lohmueller@lmu.de

Content:

S1: Synthesis of AzoLPC and d*azo*-PC.....2
S2: Photostationary states, absorbance spectra, and switching kinetics of *azo*-PC and d*azo*-PC. ...9
S3: Dynamic light scattering (DLS) experiments of d*azo*-PC pSUVs. 10
S4: Optical properties of TexasRed-DHPE and Atto633-DPPE. 11
S5: Photoswitching of *azo*-PC pSUVs with 550 nm. 11
S6: Photoluminescence measurements and photomodulation of *azo*-PC and d*azo*-PC pSUVs. 12
S7: Fluorescence intensity of photolipid-doped GUVs during green-light exposure. 13
S8: Fusion of DOPC vesicles. 13
S9: Estimation of the bending stiffness of d*azo*-PC pGUVs. 13
S10: Fluorescence recovery after photobleaching (FRAP) measurements of d*azo*-PC bilayers....14
S11: Vesicle splitting and pearling in d*azo*-PC doped GUVs. 15
S12: Domain formation in d*azo*-PC pGUVs. 15
S13: Ratiometric fluorescence analysis of charge mediated fusion. 16
References: 17

S1: Synthesis of AzoLPC and d*azo*-PC

Reagents and Instrumentation

All reagents and solvents were purchased from commercial sources (Sigma-Aldrich, TCI Europe N.V., Strem Chemicals, etc.) and were used without further purification. Solvents were obtained from Fisher Scientific. Reactions were monitored by thin layer chromatography (TLC) on pre-coated, Merck Silica gel 60 F_{254} aluminium-backed plates and the chromatograms were first visualized by UV irradiation at λ = 254 nm. Flash silica gel chromatography was performed using silica gel (SiO_2, particle size 40-63 μm) purchased from SiliCycle. NMR spectra were measured on a BRUKER Avance III HD 400 (equipped with a CryoProbe^{TM}). Multiplicities in the following experimental procedures are abbreviated as follows: s = singlet, d = doublet, t = triplet, q = quartet, m = multiplet. ^{1}H chemical shifts are expressed in parts per million (ppm, δ scale) and are referenced to the residual protium in the NMR solvent ($CDCl_3$: δ = 7.26; CD_3OD: δ = 3.31). ^{13}C chemical shifts are expressed in ppm (δ scale) and are referenced to the carbon resonance of the NMR solvent ($CDCl_3$: δ = 77.16, CD_3OD: δ = 49.00). NOTE: Due to the *trans*/*cis* isomerization of some compounds containing an azobenzene functionality, more signals were observed in the ^{1}H and ^{13}C spectra than would be expected for the pure *trans*-isomer. Only signals for the major *trans*-isomer are reported.

Synthetic Scheme for AzoLPC and d*azo*-PC

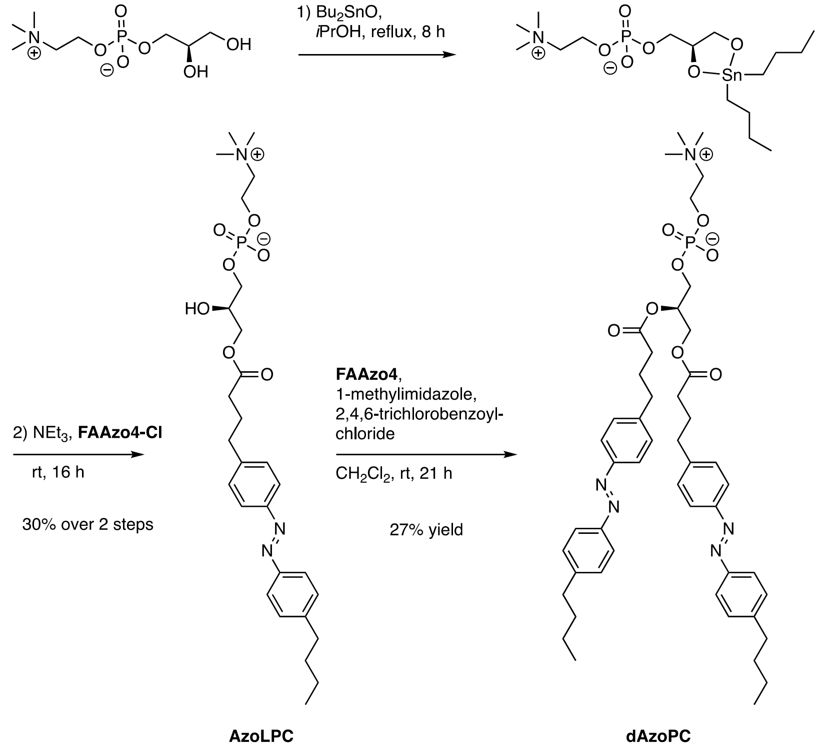

AzoLPC

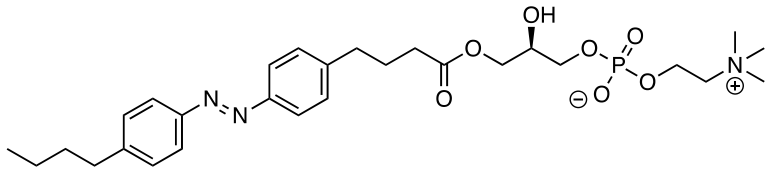

GPC (Glycerophosphocholine) (1.64 g, 6.39 mmol, 1.0 equiv.) and DBTO (dibutyltin oxide) (1.75 g, 7.03 mmol, 1.1 equiv.) were suspended in 50 mL of 2-propanol and heated to 100 °C for 8 h. After cooling the mixture to 0 °C, dry triethylamine (776 mg, 7.67 mmol, 1.2 equiv.) was added slowly. **FAAzo4-Cl** (**FAAzo4**[1] treated with excess thionyl chloride in CH_2Cl_2 (1M)) (2.63 g, 7.67 mmol, 1.2 equiv.) was dissolved in a small amount of CH_2Cl_2 and added dropwise to the reaction mixture at room temperature. The reaction mixture was stirred at room temperature under nitrogen atmosphere overnight. After removing the solvents under reduced pressure, the product was purified by flash column chromatography (CH_2Cl_2:CH_3OH (9:1), CH_2Cl_2:CH_3OH:H_2O (10:4:0.5), CH_2Cl_2:CH_3OH:H_2O (10:8:2)), which gave 1.07 g (1.90 mmol, 30 %) of **AzoLPC** as an orange solid. ^{1}H NMR (400 MHz, CD_3OD): δ (ppm) = 7.88 – 7.78 (m, 4H), 7.37 (dd, J = 12.4, 8.3 Hz, 4H), 4.33 – 4.24 (m, 2H), 4.20 (dd, J = 11.4, 4.5 Hz, 1H), 4.13 (dd, J = 11.3, 6.0 Hz, 1H), 4.03 – 3.95 (m, 1H), 3.94 – 3.86 (m, 2H), 3.65 – 3.59 (m, 2H), 3.21 (s, 9H), 2.76 (t, J = 7.7 Hz, 2H), 2.71 (t, J = 7.7 Hz, 2H), 2.42 (t, J = 7.3 Hz, 2H), 2.00 (p, J = 7.4 Hz, 2H), 1.66 (p, J = 7.6 Hz, 2H), 1.40 (sext, J = 7.4 Hz, 2H), 0.97 (t, J = 7.4 Hz, 3H). ^{13}C NMR (100 MHz, CD_3OD): δ (ppm) = 14.3, 23.4, 27.6, 34.2, 34.8, 35.9, 36.5, 54.6, 54.7, 54.7, 60.5, 66.3, 67.5, 67.8, 69.9, 123.8, 123.8, 130.2, 130.4, 146.5, 147.8, 152.3, 152.5, 174.9. ^{31}P NMR (200 MHz, CD_3OD): δ (ppm) = -50.00. HRMS: m/z calcd. for $C_{28}H_{43}N_3O_7P^+$ ($[M+H]^+$): 564.2833, found: 564.2842.

d*azo*-PC

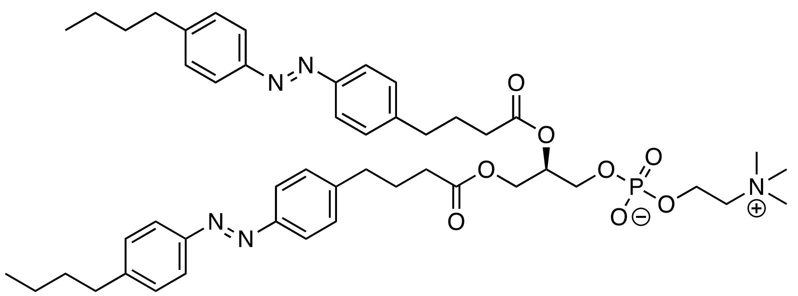

FAAzo-4 [1] (178 mg, 0.550 mmol, 2.0 equiv.) was dissolved in 5.8 mL of CH_2Cl_2. NMI (*N*-methylimidoazol) (67.7 mg, 0.825 mmol, 3.0 equiv.) was added slowly to the solution. The mixture was transferred to **AzoLPC** (155 mg, 0.275 mmol, 1.0 equiv.) in 2.9 mL of CH_2Cl_2. 2,4,6-trichlorobenzoyl chloride (201 mg, 0.825 mmol, 3.0 equiv.) was added dropwise to the mixture. The clear orange solution was stirred for 21 h at room temperature and was directly subjected to purification *via* flash column chromatography (CH_2Cl_2, CH_2Cl_2:MeOH:H_2O 99:1:0 / 95:5:0 / 8:2:0,1 / 7.5:2.5:0.1 / 7:3:0,1 / 7:3:0,2 (400 mL each) / 10:4:0.5 (600 mL)) afterwards. A second purification by flash column chromatography (CH_2Cl_2, CH_2Cl_2:MeOH:H_2O 99:1:0 / 95:5:0 / 8:2:0,1 / 7.5:2.5:0.1 / 7:3:0,2 (500 mL each) / 10:4:0.5 (1000 mL)) gave **d*azo*-PC** (64.2 mg, 0.0738 mmol, 27 %) as an orange solid. ^{1}H NMR (400 MHz, CD_3OD): δ (ppm) = 7.78 (d, J = 8.0 Hz, 8H), 7.32 (d, J = 7.7 Hz, 8H), 5.34 – 5.24 (m, 1H), 4.48 (dd, J = 12.0, 3.0 Hz, 1H), 4.31 – 4.23 (m, 2H), 4.23 – 4.16 (m, 1H), 4.09 – 3.99 (m, 2H), 3.63 – 3.58 (m, 2H), 3.19 (s, 9H), 2.75 – 2.65 (m, 8H), 2.44 – 2.33 (m, 4H), 1.95 (p, J = 6.6 Hz, 4H), 1.65 (p, J = 7.5 Hz, 4H), 1.39 (sext, J = 7.4 Hz, 4H), 0.96 (t, J = 7.4 Hz, 6H). ^{13}C NMR (100 MHz, CD_3OD): δ (ppm) = 174.5, 174.2, 152.4, 152.2, 147.8, 146.3, 130.4, 130.2, 123.9, 123.8, 71.9, 67.4, 65.0, 63.7, 60.5, 54.6, 54.6, 54.6, 36.5, 35.7, 34.8, 34.2, 27.6, 23.4, 14.3. ^{31}P NMR (162 MHz, CD_3OD): δ (ppm) = -0.56. HRMS: m/z calcd. for $C_{48}H_{65}N_5O_8P^+$ ($[M+H]^+$): 870.4565, found: 870.4577.

Figure S1: ^{1}H,^{13}C, and ^{31}P NMR Spectra

<u>AzoLPC:</u>

^{1}H NMR

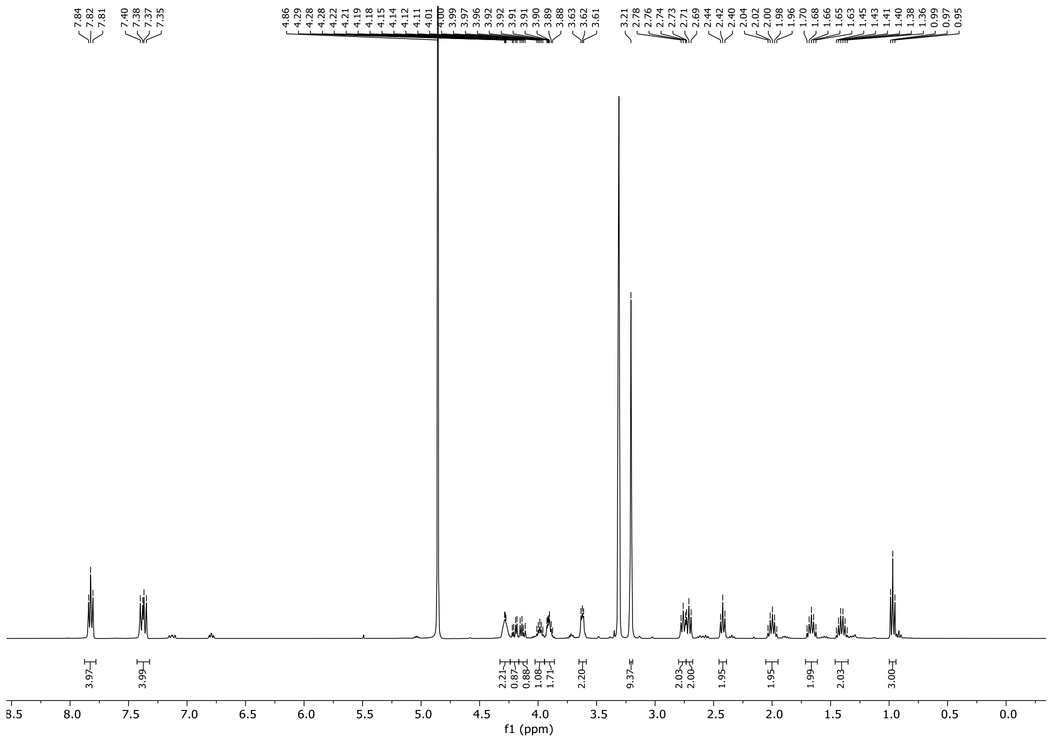

^{13}C NMR

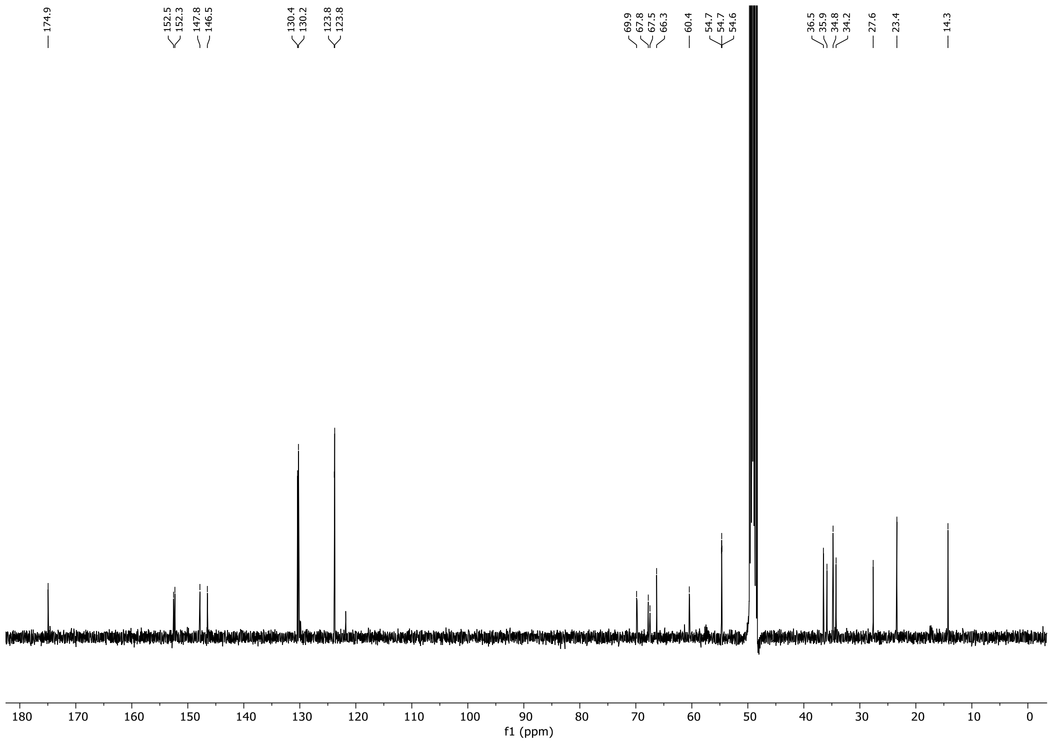

^{31}P NMR

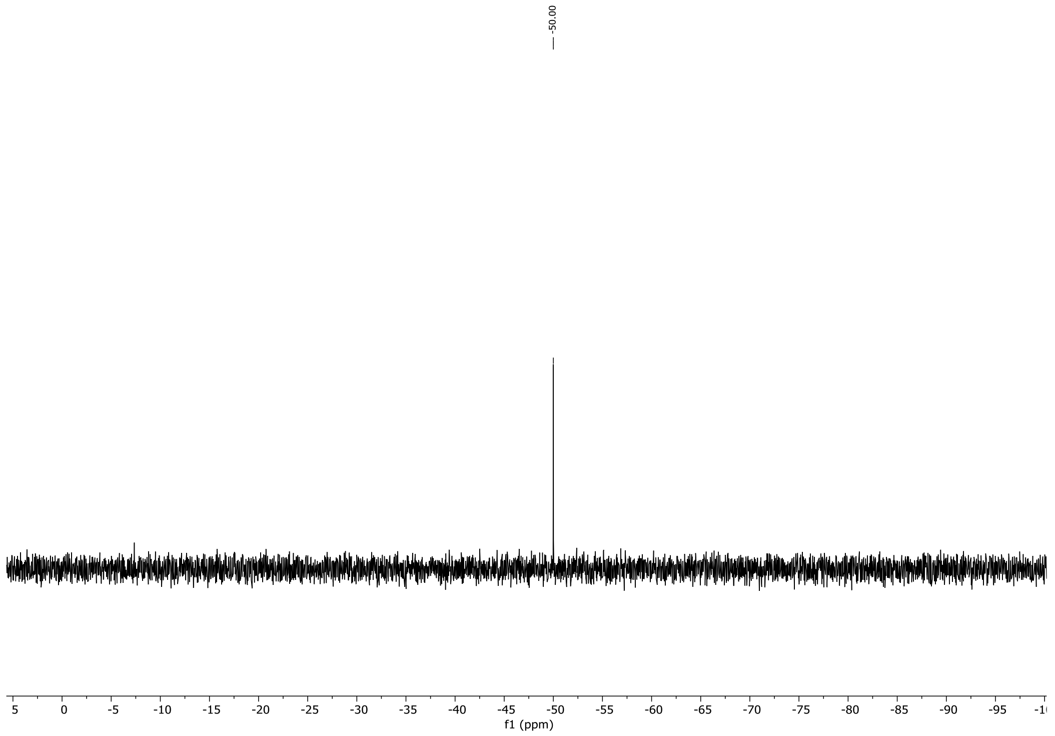

<u>d*azo*-PC:</u>

^{1}H NMR

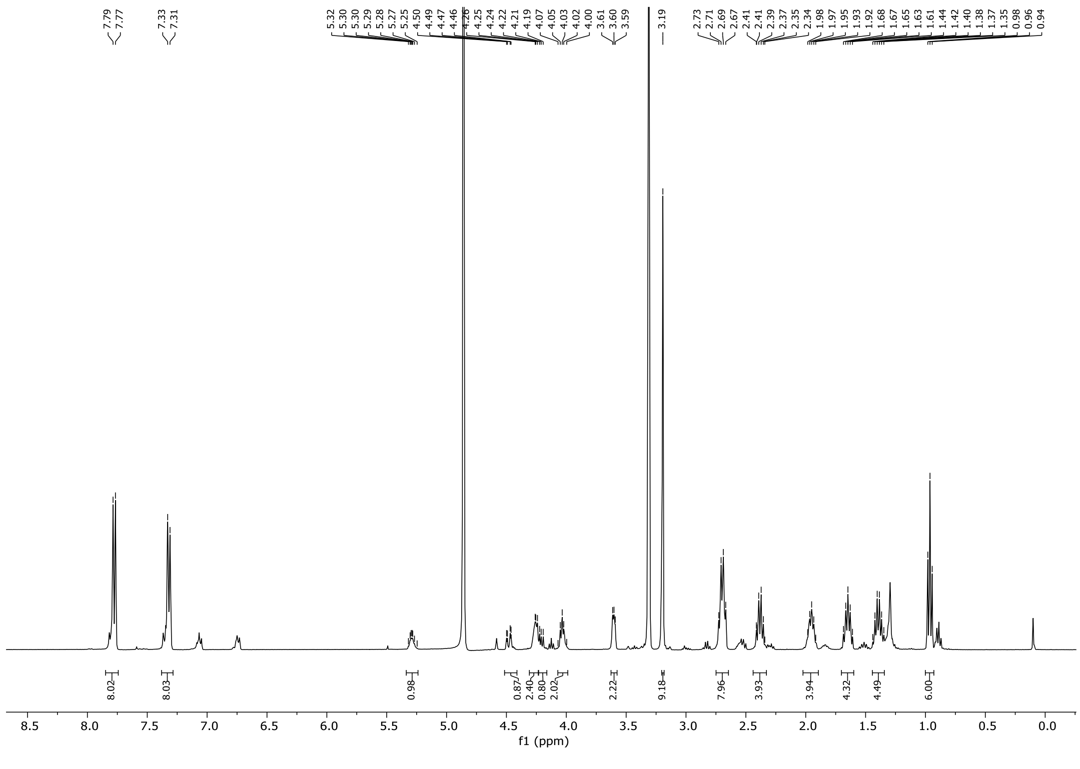

^{13}C NMR

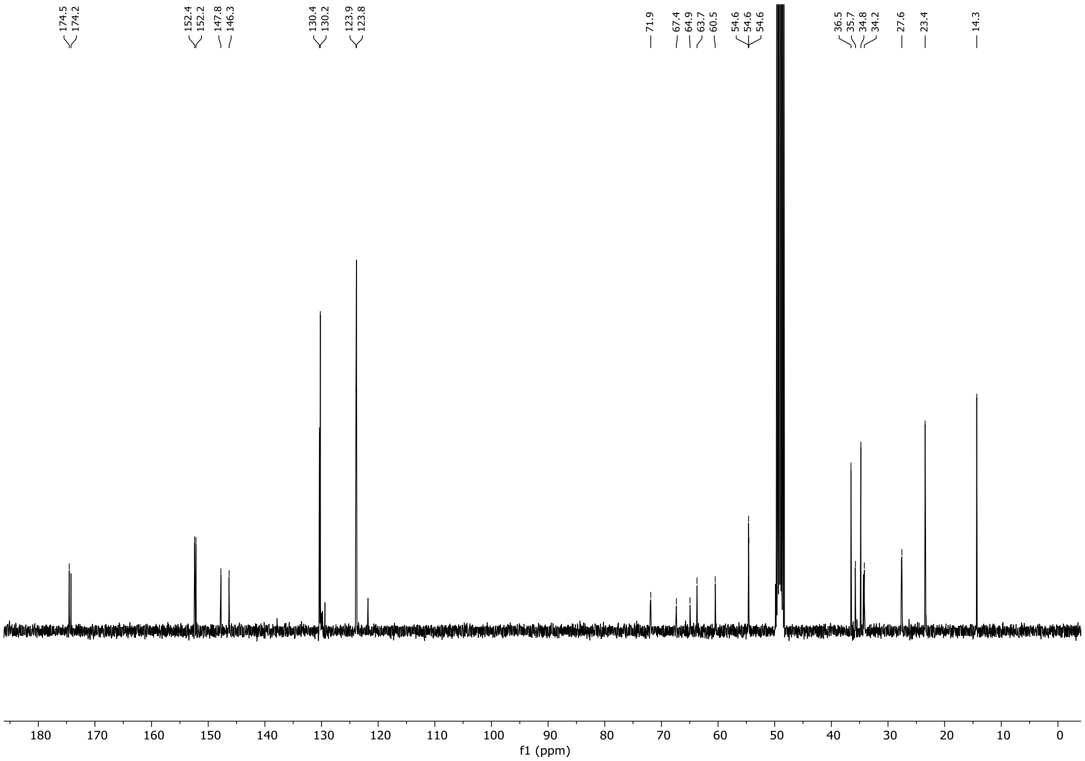

^{31}P NMR

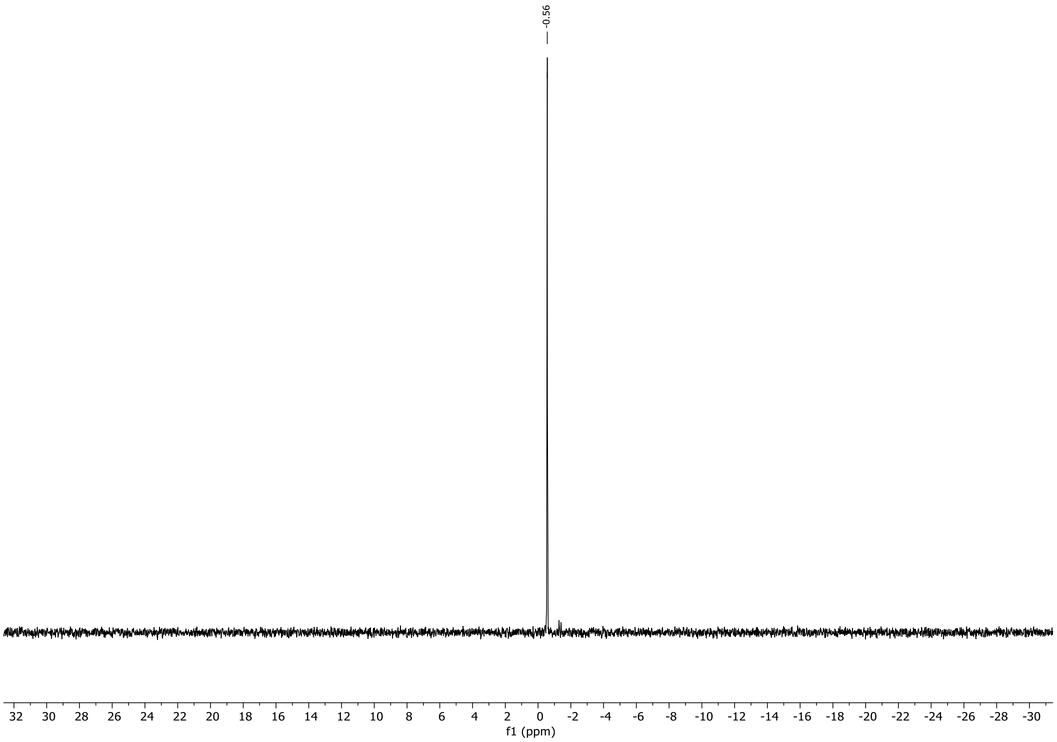

S2: Photostationary states, absorbance spectra, and switching kinetics of *azo*-PC and d*azo*-PC.

Photostationary states: To confirm that UV-A light is suitable to reach a high amount of free *cis*-**d***azo*-**PC** at the photostationary state (PSS), we determined the *trans/cis* ratios of free **d***azo*-**PC** lipids at the PSS *via* NMR measurements using a BRUKER Avance III HD 400, equipped with a CryoProbeTM (Figure S2 A). ^1H chemical shifts are expressed in parts per million (ppm) and are referenced to $CDCl_3$ as a blank. The *trans/cis* ratio at the *cis* PSS is 2:98.

Absorbance spectra: We measured the UV/vis spectra of *azo*-**PC** and **d***azo*-**PC** dissolved in chloroform and assembled to small unilamellar photolipid vesicles (pSUVs). pSUVs were made from 100 mol% photolipids. Absorbance spectra were recorded in quartz cuvettes with a UV/vis spectrophotometer (Cary 600, Agilent). Photoswitching to the photostationary *trans* and *cis* states was achieved with two fiber-coupled LEDs (365nm (P=50mW) and 465nm (P=30mW), Prizmatix) by illuminating the samples until the spectra did not change anymore (Figure S2 B). Free *azo*-**PC** and **d***azo*-**PC** lipids ($CHCl_3$) in the *trans* state show a strong absorbance at 335 nm and 338 nm, respectively, that can be attributed to the ππ* transition and a low absorbance band at ~450 nm corresponding to the nπ* band. In the *cis* state, the ππ* band shifts to 295 nm and 297 nm for *azo*-**PC** and **d***azo*-**PC**, respectively. For *trans* pSUVs the ππ* transition shifts to 315 nm and 313 nm for *azo*-**PC** and **d***azo*-**PC**, respectively. This hypsochromic shift indicates H-aggregate formation of the *trans* azobenzene groups as reported previously[2]. In the *cis* state, the maximum absorbance of the ππ* band is at 320 nm for **d***azo*-**PC**. This red-shift compared to free *cis* photolipids indicates that not all lipids were switched to the *cis* state and that the *cis* PSS still contains lipids in the *trans* state.

Switching kinetics: The switching kinetics of free *azo*-**PC** and **d***azo*-**PC** lipids (dissolved in $CHCl_3$) as well as of *azo*-**PC** and **d***azo*-**PC** pSUVs (100 mol% photolipids in ddH_2O) were determined by recording the time-lapse absorbance changes, i.e., decrease and increase (time steps: 0.01-0.1 min) of the ππ* absorbance peaks (*azo*-**PC** lipids: 335 nm; **d***azo*-**PC** lipids: 338 nm; *azo*-**PC** pSUVs: 315 nm; **d***azo*-**PC** pSUVs: 313 nm) under continuous LED irradiation (465 nm and 365 nm) with different power densities as described previously[3]. We analyzed the switching kinetics in analogy to previous reports[4-6] and determined the half-value times $T_{1/2}$ in dependence of the power density. As shown in Figure S2 C, the half-value switching times of both, *azo*-**PC** and **d***azo*-**PC** free lipids, are similar for the respective isomers. For pSUV samples, the half-value switching times of **d***azo*-**PC** vesicles are on average 2.5 times (standard deviation: 0.6) longer than the times of *azo*-**PC** vesicles. In addition, the half-value switching times decrease with increasing LED power densities.

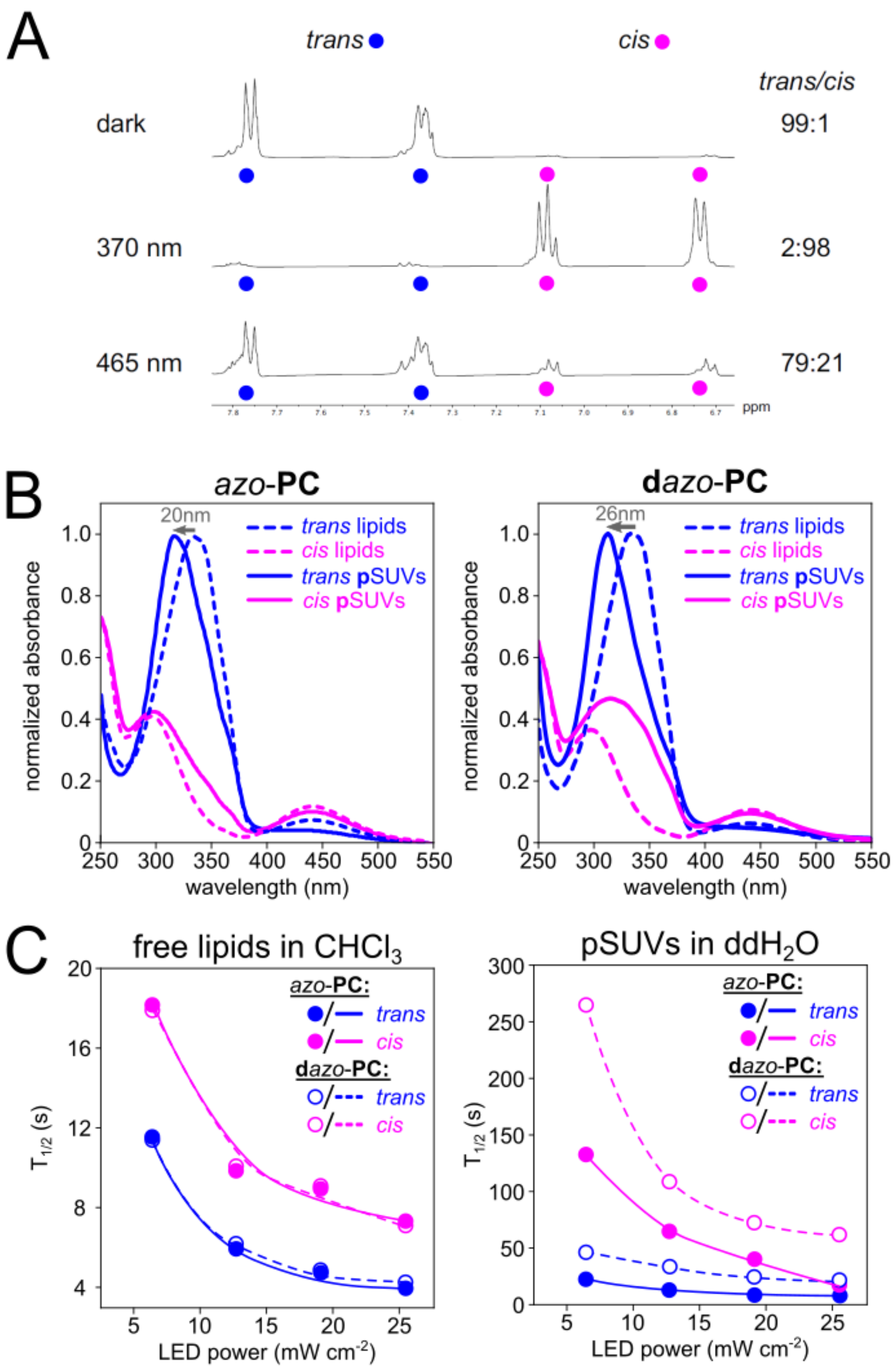

Figure S2: (**A**) NMR spectra of **d***azo*-**PC** in $CDCl_3$. The *trans/cis* ratios are 99:1 in the dark state, 79:21 in the *trans* state, and 2:98 in the *cis* state. (**B**) Absorbance spectra of *azo*-**PC** and **d***azo*-**PC** lipids and pSUVs. Free lipids in the *trans* state (dashed blue lines) show a strong absorbance (ππ* transition) at 335 nm and 338 nm for *azo*-**PC** and **d***azo*-**PC**, respectively, and a low absorbance (nπ* transition) at ~450 nm. The *cis* spectra (dashed purple lines) exhibit a strong ππ* absorbance band at 295 nm (*azo*-**PC**) and at 297 nm (**d***azo*-**PC**), and a low intensity nπ*band at ~450 nm. For *azo*-**PC** and **d***azo*-**PC** pSUVs in the *trans* state, the ππ* transitions are blue-shifted by 20 nm and 26 nm to 315 nm and 313 nm, respectively. In the *cis* state, the maximum ππ* transition is at 320 nm for the **d***azo*-**PC** pSUV sample. **(C)** Half-value switching times ($T_{1/2}$) of *azo*-**PC** and **d***azo*-**PC** lipids in $CHCl_3$ (left) and *azo*-**PC** and **d***azo*-**PC** pSUVs (right).

S3: Dynamic light scattering (DLS) experiments of d*azo*-PC pSUVs.

DLS measurements were done with a Zetasizer Nano ZS device (Malvern Panalytics). Quartz cuvettes were used to determine the size distribution of **d***azo*-**PC** pSUVs (100 mol% **d***azo*-**PC** in ddH_2O). Prior data acquisition, the vesicle samples were illuminated with fiber-coupled LEDs (365 nm or 465 nm, Prizmatix) for ~5 min, to switch between the *trans* and *cis* states. For pSUVs in the *trans* state, we found an average

pSUV diameter of 246 nm (STD = 6 nm) that increases to 313 nm (STD = 11 nm) upon *trans*-to-*cis* isomerization (Figure S3). The data points were derived from four consecutive measurements of the same vesicle sample. The error bars represent the standard deviation of the single measurements.

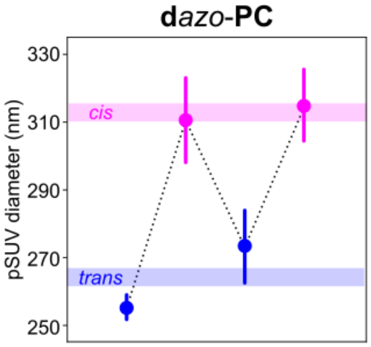

Figure S3: The diameters of **d***azo***-PC** pSUVs are larger for photolipids in the *cis* state than for *trans* isomers.

S4: Optical properties of TexasRed-DHPE and Atto633-DPPE.

The absorbance and photoluminescence (PL, Figure S4) spectra were obtained from dye-labeled lipids dissolved in chloroform (amylene stabilized, Sigma Aldrich). UV/vis and PL measurements were performed with a UV/vis spectrophotometer (Carry 600, Agilent) and a Fluorolog-3 FL3-22 spectrometer (Horiba Scientific, see S6 for details) using quartz cuvettes.

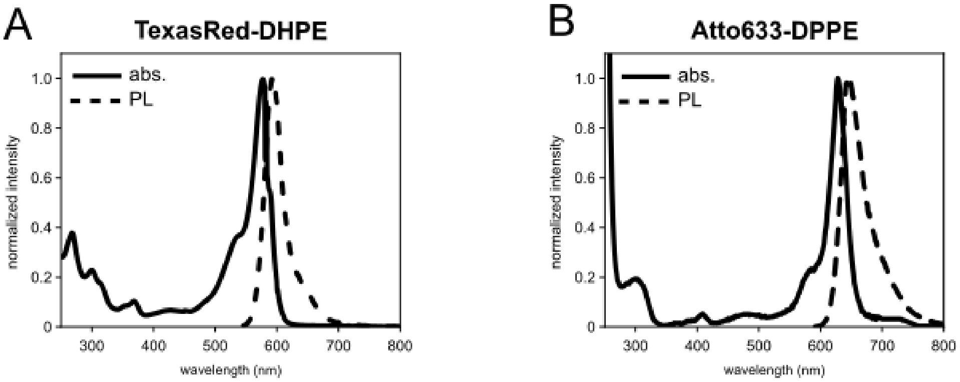

Figure S4: Absorbance and PL spectra of TexasRed-DHPE (**A**) and Atto633-DPPE (**B**).

S5: Photoswitching of *azo*-PC pSUVs with 550 nm.

The photoswitching efficiency of *azo*-**PC** pSUVs with green light of 550 nm was studied *via* absorbance measurements. A lipid sample was illuminated with fiber-coupled LEDs (365nm with P_{max} = 75mW, 465nm with P_{max} = 80mW, 550nm with P_{max} = 20mW, Prizmatix) to induce photoswitching. Intermediate *trans/cis* states were recorded for 550nm-exposure. As shown in Figure S5, green light of 550 nm is sufficient to

recoup the *trans* absorbance reachable with 465 nm. The longer switching time of 11 min can be explained by the lower absorbance and hence switching efficiency of the pSUVs at 550 nm compared to 465 nm and the lower LED power, i.e., 20 mW (550nm) compared to the 80 mW of the 465nm-LED.

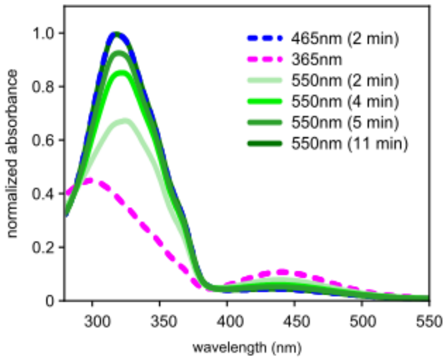

Figure S5: Absorbance spectra of *azo*-**PC** pSUVs using 365 nm (P_{max} = 75 mW), 465 nm (P_{max} = 80 mW), and 550 nm (P_{max} = 20mW).

S6: Photoluminescence measurements and photomodulation of *azo*-PC and d*azo*-PC pSUVs.

Photoluminescence (PL) spectra were recorded with a Fluorolog-3 FL3-22 spectrometer (Horiba Scientific) equipped with a 450W Xenon lamp, double monochromators for both excitation and emission, and a water-cooled R928 photomultiplier tube. The spectra were corrected with respect to the spectral sensitivity of the detector and the excitation intensities. Photomodulation was studied by PL measurements of pSUVs containing 99 mol% of photolipids and 1 mol% TexasRed-DHPE after excitation with 550 nm. For both samples, the TexasRed-emission is higher for *trans* than for *cis* pSUVs (Figure S6). Impacts of the photolipid absorption coefficients have been neglected, because the *cis* and *trans* absorbances at 550 nm are both very low (Figure S2 B). The photomodulation efficiencies (E_{mod}) were calculated according to $E_{mod} = 1 - \frac{I_{cis}}{I_{trans}}$[7] with I_{cis} and I_{trans} being the maximum PL intensities of the *trans* and *cis* pSUVs, respectively, and are E_{mod}^{azo-PC}= 79% and $E_{mod}^{\mathbf{d}azo-PC}$ = 72%.

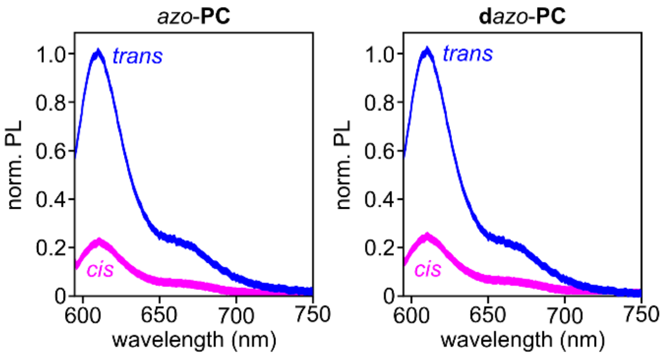

Figure S6: PL spectra of *azo*-**PC** (left) and **d***azo*-**PC** (right) pSUVs labeled with 1 mol% TexasRed-DHPE. For both samples, the dye emission is higher in presence of *trans* photolipids compared to *cis* isomers.

S7: Fluorescence intensity of photolipid-doped GUVs during green-light exposure.

After mixing pSUVs (made from 96 mol% *azo*-**PC** or **d***azo*-**PC**, 3 mol% DOTAP, and 1 mol% TexasRed-DHPE) and DOPC GUVs (made from 100 mol% DOPC) and subsequent vesicle fusion, we imaged the samples with green light (510-550 nm) for several seconds and analyzed the time-lapse PL intensities. As shown in Figure S7, the intensities decrease slightly (~7-10%) over time for both photolipids due to photobleaching.

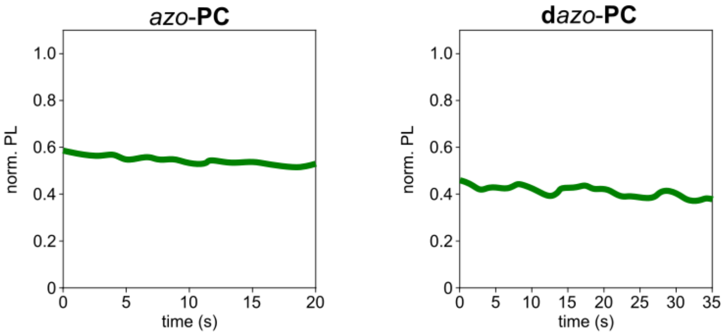

Figure S7: PL intensities of GUVs after fusion with TexasRed-labeled pSUVs during green-light illumination.

S8: Fusion of DOPC vesicles.

DOPC SUVs were prepared from 96 mol% DOPC, 3 mol% DOTAP, and 1 mol% TexasRed-DHPE. DOPC GUVs were made from 95 mol% DOPC and 5 mol% PA. After mixing of the vesicle samples and subsequent vesicle fusion, the GUV became fluorescent and detectable *via* fluorescence microscopy (Figure S8).

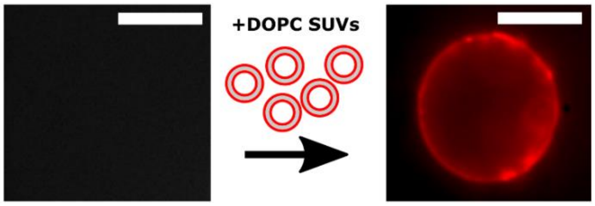

Figure S8: Fusion of red-fluorescent, cationic DOPC SUVs and non-fluorescent, anionic DOPC GUVs. The GUV was imaged *via* epifluorescence using green light (510-550nm). Scale bars: 10µm.

S9: Estimation of the bending stiffness of d*azo*-PC pGUVs.

The bending stiffness values of **d***azo*-**PC** pGUVs containing 1 mol% TexasRed-DHPE were estimated *via* time-lapse flickering spectroscopy using our previously reported protocol[8]. Image series (exposure time: 0.1 s) were acquired with a CCD camera (iXon Ultra, Andor) and analyzed based on the method by Häckl et al.[9] (further details can also be found in ref.[8]). The vesicle contours were recorded in epifluorescence mode using green and UV-A light (Figure S9). Hence, we could not fully avoid photobleaching in order to get sufficiently intense contour images. We were thus restricted to total times of $t_0 = (5 \pm 3)s$ (# frames:

20-100; frame rate: 10 s^{-1}). To account for this resolution mismatch, we analyzed the full contours (360°) of 10 different vesicles with diameters of ~8-22 µm and determined the bending stiffness from the average. We quantified the bending stiffness of several pGUVs under green and UV-A light exposure. The bending stiffness values are summarized in Table S1.

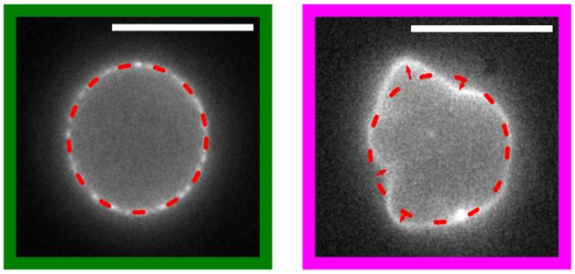

Figure S9: Epi-fluorescence images of **d***azo***-PC** pGUVs labeled with 1 mol% TexasRed-DHPE under green (510-550nm, green box) and UV-A (330-385nm, pink box) light exposure. The red dotted lines represent the reference circle fitted to the pGUV contour. The red arrows highlight the deviations from the circle, i.e., the membrane fluctuations. Scale bars: 10µm.

green light (510-550nm) - *trans*		UV-A light (330-385nm) - *cis*	
κ (10^{-17} J)	pGUV diameter (µm)	κ (10^{-20} J)	pGUV diameter (µm)
1.2±0.9	13.4	0.8±0.7	13.4
2.5±2.1	13.6	1.7±1.5	13.5
0.5±0.3	12.3	5.5±5.4	12.3
0.7±0.5	11.7	0.2±0.1	11.7
4.3±3.9	9.0	2.0±1.8	9.0
8.2±7.0	9.3	0.22±0.19	9.3
0.29±0.26	20.1	1.3±0.9	20.1
0.87±0.76	8.3	0.21±0.15	8.3
1.9±1.5	17.5	3.2±2.5	17.5
2.9±2.8	22.3	6.3±5.2	22.3

Table S1: Kappa Values of **d***azo***-PC** pGUVs.

S10: Fluorescence recovery after photobleaching (FRAP) measurements of d*azo*-PC bilayers.

Supported **d***azo***-PC** bilayers were prepared by drop casting TR-labeled (1 mol% TR-DHPE) pSUVs on clean glass slides (cleaned *via* sonication in acetone, propanol, and ddH_2O each for 5 min) and inducing vesicle fusion and subsequent bilayer formation[10]. Excess pSUVs were removed *via* multiple rinsing steps. Photoswitching and imaging were achieved simultaneously using UV-A (330-385 nm) and green (510-550 nm) light (see experimental section for further details on the microscope setup). For FRAP, a small spot of the bilayer membrane was bleached using intense green light. The fluorescence recoveries were determined *via* acquisition of 100-200 image frames (exposure times: 0.1-0.2 s) and analyzed according to the protocol by Jönsson et al.[11] As illustrated in Figure S10, the average diffusion coefficients change between D_{trans} = (0.11 ± 0.01) µm^{2}s^{-1} and D_{cis} = (1.3 ± 0.1 µm^{2}s^{-1}).

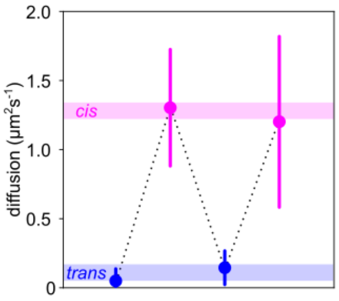

Figure S10: Reversible switching of diffusion coefficients of *cis*-**d***azo*-**PC** and *trans*-**d***azo*-**PC** bilayer membranes.

S11: Vesicle splitting and pearling in d*azo*-PC doped GUVs.

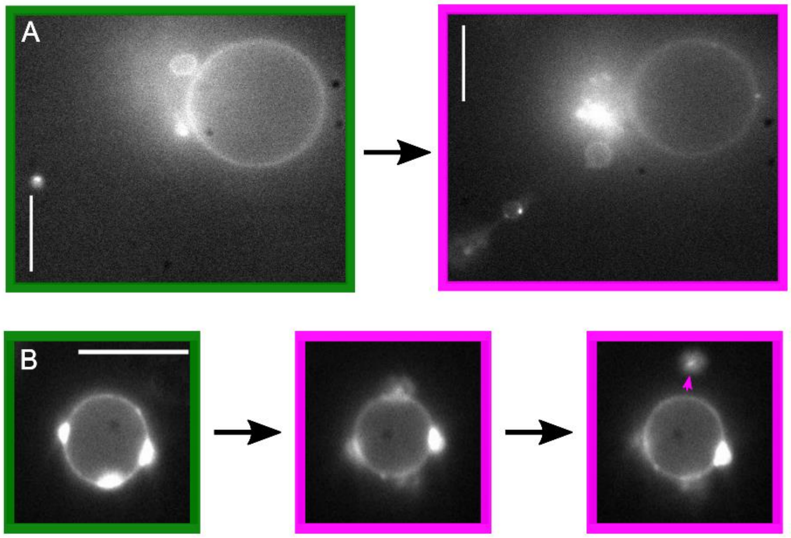

Figure S11: (**A**) Pearling transition of **d***azo*-**PC**-doped GUV upon UV-A exposure (pink box). (**B**) Vesicle splitting of **d***azo*-**PC**-doped GUV upon UV-A exposure. The small daughter vesicle is marked by the pink arrow. Green boxes: 510-550 nm. Pink boxes: 330-385 nm. Scale bars: 10 µm.

S12: Domain formation in d*azo*-PC pGUVs.

The **d***azo*-**PC** GUVs were made from 99 mol% **d***azo*-**PC** and 1 mol% TexasRed-DHPE *via* electroformation. The GUVs in the *trans* state were imaged with green light (Figure S12). They display bright and dark areas indicative for phase separation. During UV illumination, which switches the lipids to *cis*, the domains disappear and the vesicle contour starts to immediately fluctuate.

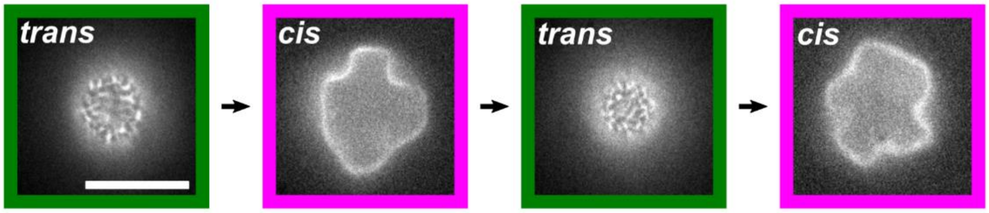

Figure S12: Domain formation in **d***azo*-**PC** pGUVs. Bright and dark areas are visible on the pGUV during green-light exposure (green boxes, 510-550 nm). Membrane fluctuations arise upon UV-A exposure (pink boxes, 330-385 nm). Scale bar: 10 µm.

S13: Ratiometric fluorescence analysis of charge mediated fusion.

Green-fluorescent, anionic GUVs were made from 94 mol% DOPC, 1 mol% Atto465-DOPE, and 5 mol% PA and imaged *via* epifluorescence microscopy. They show a green color during illumination with blue light and no emission during green-light exposure (Figure S13 A). We then added red-fluorescent pSUVs made from 3 mol% DOTAP, 1 mol% TexasRed, and 96 mol% photolipids (*azo*-**PC** or **d***azo*-**PC**). After mixing and subsequent vesicle fusion, the GUV changed its color from green to red upon uptake of *azo*-**PC** during blue light exposure and showed a red-fluorescence upon green-light exposure (Figure S13 B). For **d***azo*-**PC**, the GUV maintained a predominant green color (Figure S13 C). However, after switching to green-light illumination the GUV showed a red color, indicative for the uptake of the red-fluorescent **d***azo*-**PC** pSUVs. We then analyzed the red and green (RG) intensities of the GUVs and calculated the intensity ratios of the two emission channels of the vesicle contours. These values were compared to calibration curves, derived from the PL spectra of SUVs at which the lipids were already added during the liposome preparation (Figure S13 D). These SUVs contained defined amounts of all lipids, i.e., DOTAP, PA, TexasRed-DHPE, Atto465-DOPE, DOPC, and *azo*-**PC** or **d***azo*-**PC**. To mimic fusion, the amount of DOPC, PA, and Atto465-DOPE, which represent the green-fluorescent, anionic GUVs was kept constant for all samples (6.36 mM, 100 µL of lipid solution containing 94 mol% DOPC, 5 mol% PA, and 1 mol% Atto465-DOPE). Instead, the amount of lipids representing the pSUVs, i.e., 96 mol% *azo*-**PC** or **d***azo*-**PC**, 3 mol% DOTAP, and 1 mol% TexasRed-DHPE (6.36 mM), was increased from 10-90 mol% (11.1-150 µL) with respect to the lipid volume containing DOPC, Atto465-DOPE and PA. The PL spectra were obtained after excitation with 465 nm. Finally, the ratios of the PL intensities at 502 nm and 610 nm were calculated for both photolipids (Figure S13 E, F). The red crosses represent the RG ratios determined from the GUVs after vesicle fusion. From the molar ratios of the two lipid dyes the DOPC/photolipid ratios can be derived, since they increase simultaneously. They are 3.4 ± 2.4 and 5.7 ± 3.3, corresponding to photolipid contents, i.e., fusion efficiencies of ~ 40 ± 30 % and ~ 20 ± 15 % for *azo*-**PC** and **d***azo*-**PC**, respectively. To further support these values, we also analyzed the size increase of the GUVs upon vesicle fusion (Figure S13 G). The line plots illustrate an increase of the GUV size by 0.2 µm, i.e., 8 % within 4 s.

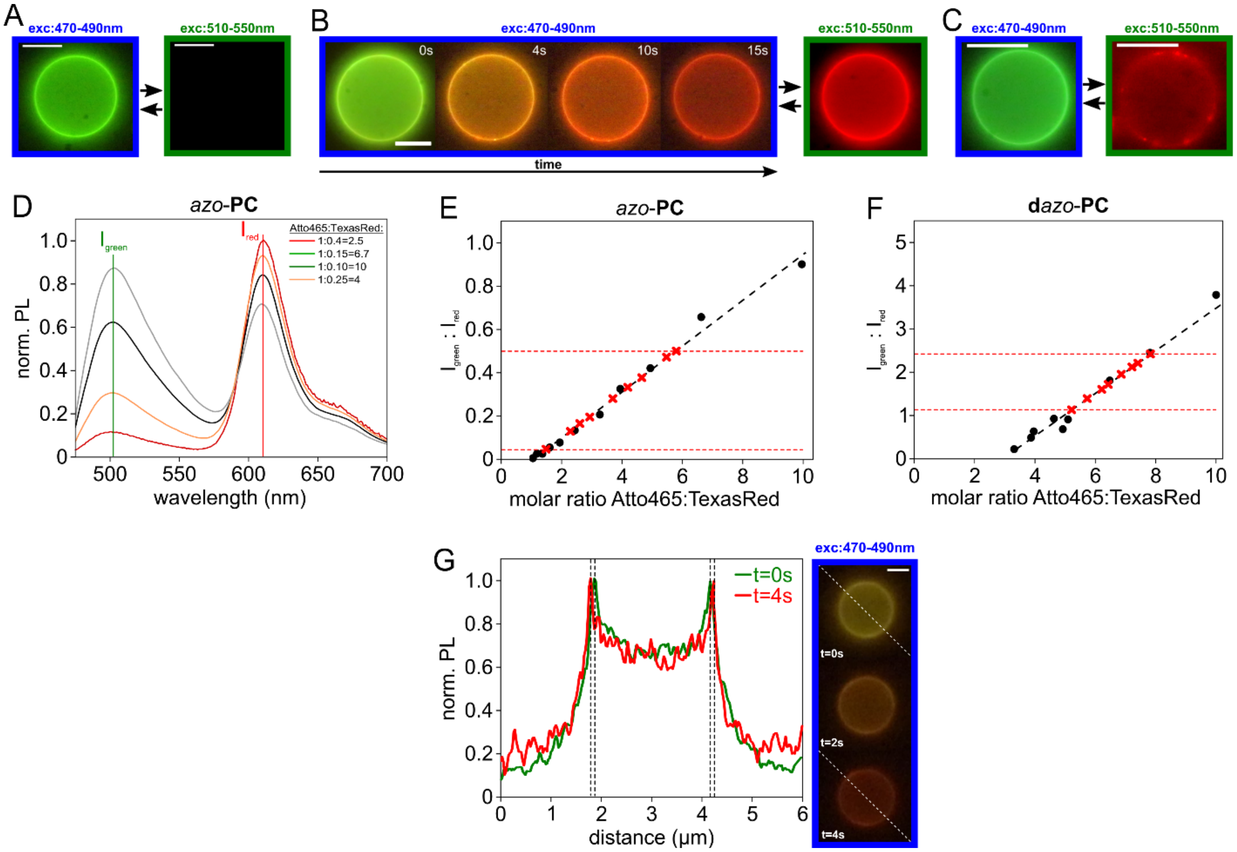

Figure S13. Fluorescence fusion assay. (**A**) Fluorescence image of green-fluorescent, anionic, DOPC GUV during blue (470-490nm) and green (510-550nm) illumination. (**B**) The DOPC GUV changes its color from green to red within 15 s during blue-light illumination upon uptake of *azo*-**PC** pSUVs and shows a red color during green-light exposure. (**C**) The DOPC GUV shows a green and a red color during blue- and green-light illumination, respectively, after mixing with **d***azo*-**PC** pSUVs. (**D**) PL spectra of SUVs containing DOPC, *azo*-**PC** or **d***azo*-**PC**, DOTAP, PA, TexasRed-DHPE and Atto465-DOPE of different molar ratios. (**E,F**) Intensity ratios (Igreen:Ired) of *azo*-**PC**-containing (**E**) and **d***azo*-**PC**-containing (**F**) pSUVs with different Atto465:TexasRed ratios. The red crosses represent the RG ratios derived from GUV images after vesicle fusion. Scale bars: 10 µm. (**G**) Size increase of DOPC GUV upon *azo*-**PC** pSUV fusion. Scale bars: 10 µm.

References:

1. Frank, J. A.; Moroni, M.; Moshourab, R.; Sumser, M.; Lewin, G. R.; Trauner, D., Photoswitchable fatty acids enable optical control of TRPV1. *Nat. Commun.* **2015,** *6*, 7118-7118.
2. Urban, P.; Pritzl, S. D.; Konrad, D. B.; Frank, J. A.; Pernpeintner, C.; Roeske, C. R.; Trauner, D.; Lohmüller, T., Light-Controlled Lipid Interaction and Membrane Organization in Photolipid Bilayer Vesicles. *Langmuir* **2018,** *34* (44), 13368-13374.
3. Pritzl, S. D.; Urban, P.; Prasselsperger, A.; Konrad, D. B.; Frank, J. A.; Trauner, D.; Lohmüller, T., Photolipid Bilayer Permeability is Controlled by Transient Pore Formation. *Langmuir* **2020,** *36* (45), 13509-13515.
4. Arya, P.; Jelken, J.; Lomadze, N.; Santer, S.; Bekir, M., Kinetics of photo-isomerization of azobenzene containing surfactants. *J. Chem. Phys.* **2020,** *152* (2), 024904.
5. Weber, C.; Liebig, T.; Gensler, M.; Zykov, A.; Pithan, L.; Rabe, J. P.; Hecht, S.; Bléger, D.; Kowarik, S., Cooperative Switching in Nanofibers of Azobenzene Oligomers. *Sci. Rep.* **2016,** *6* (1), 25605.

6. Döbbelin, M.; Ciesielski, A.; Haar, S.; Osella, S.; Bruna, M.; Minoia, A.; Grisanti, L.; Mosciatti, T.; Richard, F.; Prasetyanto, E. A.; De Cola, L.; Palermo, V.; Mazzaro, R.; Morandi, V.; Lazzaroni, R.; Ferrari, A. C.; Beljonne, D.; Samorì, P., Light-enhanced liquid-phase exfoliation and current photoswitching in graphene–azobenzene composites. *Nat. Commun.* **2016,** *7* (1), 11090.
7. Harbron, E. J., Fluorescence Intensity Modulation in Photochromic Conjugated Polymer Systems. *Isr. J. Chem.* **2013,** *53* (5), 256-266.
8. Pritzl, S. D.; Konrad, D. B.; Ober, M. F.; Richter, A. F.; Frank, J. A.; Nickel, B.; Trauner, D.; Lohmüller, T., Optical Membrane Control with Red Light Enabled by Red-Shifted Photolipids. *Langmuir* **2021**.
9. Häckl, W.; Seifert, U.; Sackmann, E., Effects of Fully and Partially Solubilized Amphiphiles on Bilayer Bending Stiffness and Temperature Dependence of the Effective Tension of Giant Vesicles. *J. phys.* **1997,** *7* (8), 1141-1157.
10. Urban, P.; Pritzl, S. D.; Ober, M. F.; Dirscherl, C. F.; Pernpeintner, C.; Konrad, D. B.; Frank, J. A.; Trauner, D.; Nickel, B.; Lohmueller, T., A lipid photoswitch controls fluidity in supported bilayer membranes. *Langmuir* **2020,** *36* (10), 2629-2634.
11. Jönsson, P.; Jonsson, M. P.; Tegenfeldt, J. O.; Höök, F., A method improving the accuracy of fluorescence recovery after photobleaching analysis. *Biophys. J.* **2008,** *95* (11), 5334-5348.
